# Immunologic barriers in liver transplantation: a single-cell analysis of the role of mesenchymal stem cells

**DOI:** 10.3389/fimmu.2023.1274982

**Published:** 2023-12-07

**Authors:** Haitao Li, Saihua Yu, Haiyan Liu, Lihong Chen, Hongzhi Liu, Xingwen Liu, Conglong Shen

**Affiliations:** ^1^Department of Hepatopancreatobiliary Surgery, Mengchao Hepatobiliary Hospital of Fujian Medical University, Fuzhou, China; ^2^College of Biological Science and Engineering, Fuzhou University, Fuzhou, China; ^3^Department of Pathology, Mengchao Hepatobiliary Hospital of Fujian Medical University, Fuzhou, China; ^4^Department of Nursing, Mengchao Hepatobiliary Hospital of Fujian Medical University, Fuzhou, China

**Keywords:** liver transplantation, immune cells, allograft tolerance, MSCs, immunosuppressant

## Abstract

**Background:**

This study aimed to analyze the biomarkers that may reliably indicate rejection or tolerance and the mechanism that underlie the induction and maintenance of liver transplantation (LT) tolerance related to immunosuppressant or mesenchymal stem cells (MSCs).

**Methods:**

LT models of Lewis-Lewis and F344-Lewis rats were established. Lewis-Lewis rats model served as a control (Syn). F344-Lewis rats were treated with immunosuppressant alone (Allo+IS) or in combination with MSCs (Allo+IS+MSCs). Intrahepatic cell composition particularly immune cells was compared between the groups by single-cell sequencing. Analysis of subclusters, KEGG pathway analysis, and pseudotime trajectory analysis were performed to explore the potential immunoregulatory mechanisms of immunosuppressant alone or combined with MSCs.

**Results:**

Immunosuppressants alone or combined with MSCs increases the liver tolerance, to a certain extent. Single-cell sequencing identified intrahepatic cell composition signature, including cell subpopulations of B cells, cholangiocytes, endothelial cells, erythrocytes, hepatic stellate cells, hepatocytes, mononuclear phagocytes, neutrophils, T cells, and plasmacytoid dendritic cells. Immunosuppressant particularly its combination with MSCs altered the landscape of intrahepatic cells in transplanted livers, as well as gene expression patterns in immune cells. MSCs may be included in the differentiation of T cells, classical monocytes, and non-classical monocytes.

**Conclusion:**

These findings provided novel insights for better understanding the heterogeneity and biological functions of intrahepatic immune cells after LT treated by IS alone or in combination with MSCs. The identified markers of immune cells may serve as the immunotherapeutic targets for MSC treatment of liver transplant rejection.

## 1 Introduction

Liver transplantation (LT) remains the standard treatment option for decompensated end-stage liver disease, acute fulminant liver failure, and even primary malignancy (1). In liver transplant patients, spontaneous acceptance of liver allografts is relatively rare although rejection is relatively easy to reverse. Tolerance is a fundamental and intrinsic component of immunity, which allows for recognition of specific antigens and subsequent immunoregulation achieved through central and peripheral mechanisms (2). The mechanisms of rejection of liver transplants may differ in degrees and cellular involvement (3). Liver-specific cell populations, such as Kupffer cells (KCs), liver sinusoidal epithelial cells, and hepatic stellate cells, may contribute to liver tolerogenicity (3). Other mechanisms, such as microchimerism, soluble major histocompatibility complex, donor human leukocyte antigen-C genotype, and regulatory T cells, may participate in inducing tolerance (4–7).

Over the years, short-term clinical outcomes after transplantation have improved due to advances in immunosuppressive therapies that have reduced the incidence of acute and chronic rejection (8, 9). The triple-drug immunosuppressive regimen remains the currently accepted standard immunosuppression for LT, based on the calcineurin inhibitor tacrolimus, short-term steroids, and antimetabolites mofetil mycophenolate or azathioprine (10, 11). However, this therapeutic regimen needs to be challenged given the long-term side effects resulting from chronic immunosuppression, the evolving definition of rejection, and the customization of the immunosuppressive load (10, 11). Particularly, the long-term clinical use of immunosuppressants has led to concerns about the emergence of adverse events such as organ toxicity, increased risk of infection, metabolic disorders and malignancy (12, 13). To minimize or withdraw immunosuppressive requirements and avoid allograft loss or failure, diligent efforts have been made to reduce the high morbidity of chronic immunosuppressive therapy. Therefore, ascertaining specific and sensitive predictors of tolerance induction or immunosuppression discontinuation will move the filed forward toward the target of facilitating long-term allograft survival without immunosuppression (14, 15). Cell-based immunotherapy can induce host tolerance to transplanted organs and significantly prolong immunosuppression, involving no nonspecific immunosuppression in LT (16).

Adoptive transfer of various cell products is applied to immune cell therapy, which has been confirmed to be well tolerated and feasible in early-phase clinical trials (17). Mesenchymal stem cells (MSCs) have recently emerged as promising candidates for cell-based immunotherapy promoting tolerance of solid allografts because they modulate the immune response (18). MSCs possess regenerative potential and are involved in the regeneration of marginal organs after LT, and therefore, are able to improve overall clinical outcome (19). Additionally, MSCs regulate hematopoiesis and the engraftment of transplanted hematopoietic stem cells in animal models by secreting cytokines and growth factors (20). With respect to suppressing T cell proliferation in a clinically significant way, MSCs compete with other cell populations (21). Mainly antiproliferative effects were detected when MSCs were cultured with lymphocytes, which may be exploited to protect solid organ grafts from being rejected (22). In addition, the functional mechanism of MSCs in combination with immunosuppressants needs to be further elucidated.

To date, biomarker studies have been relatively comprehensive, ranging from flow cytometry data about the specific immune cell subsets to transcriptome analysis, which has helped to identify genotypic or phenotypic features that favor operational tolerance after LT (23, 24). Determining the local and systemic immune phenotype of surgically tolerant transplant patients and elucidating the mechanisms by which tolerance is achieved are important goals of current tolerance studies after LT. With the establishment of more sophisticated gene expression assays, researchers developed multigene panels on this basis to identify potentially tolerogenic molecules in peripheral blood with high predictive accuracy (25). By profiling the cell types and immune markers after LT with application of immunosuppressants or MSCs, it is possible to understand the mechanism of liver transplant rejection/tolerance. Additionally, the availability of better immune monitoring could help developing strategies to recognize tolerance and reduce rejection.

Rodent LT can provide important information about immunological events and immunological mechanisms. Rodent studies, including the use of surgically demanding mouse orthotopic liver transplant model, in which major histocompatibility complex (MHC)-mismatched grafts are accepted without immunosuppressive treatment, have enabled the use of genetically modified donors and/or recipients for mechanistic studies. Using rat orthotopic liver transplant model, this study aimed to analyze the biomarkers that may reliably indicate rejection or tolerance and the mechanism that underlie the induction and maintenance of liver transplant tolerance related to immunosuppressant or MSCs.

## 2 Materials and methods

### 2.1 Animals

Specific pathogen-free male F344 rats (weight, 50-70 g; age, 3 weeks) were the sources of MSCs. Male Lewis rats (weight, 275-285 g; age, 8 weeks) were used as recipients and syngeneic donors (Syn). Male F344 rats (weight, 260-270 g; age 8 weeks) were used as allogeneic donors. F344 rat bone marrow (BM)-derived MSCs have strong proliferation and multi-directional differentiation capabilities, which can be used for studying proliferation, aging, immunity, differentiation and transplantation. Fischer 344 rats became a favorite strain for studying tumor transplantation, carcinogenicity, aging, toxicology and other general research. Numerous amounts of studies used male F344 rats as donors in rat LT (26, 27). Lewis rat strains are inbred rat strains, suitable for research on transplants between major histocompatibility complex (MHC)-mismatched strains (28). All animals were purchased from Beijing Vital River Co [SCXK (jin) 2021-0006]. The rats were housed in temperature- and humidity-controlled animal facilities under a 12-h light/dark cycle for at least one week prior to surgery under standard conditions. The animals were fed a standard diet and tap water *ad libitum*. The animal study was approved by Animal Ethics Committee of Mengchao Hepatobiliary Hospital of Fujian Medical University (NO. MCHH-AEC-2023-04-01). The study was conducted in accordance with the local legislation and institutional requirements. All experimental procedures were carried out according to the Health Care and Use Guidelines of Laboratory Animals (8th edition) (29).

### 2.2 Isolation and culture of MSCs

F344 rats were anesthetized with 1% pentobarbital sodium and then sacrificed by cervical dislocation. Under sterile conditions, the bilateral tibia and femur were collected, and soaked in a sterile PBS (Gibco, USA). After excision of the both ends of the bone, a 1.0 mL syringe containing α-MEM (Hyclone, USA) was used to repeatedly wash the bone marrow cavity. Afterwards, the collected solutions were filtered through a cell strainer (100 μm), followed by eliminating the red blood cells with osmotic lysates (Beyotime biotechnology, Shanghai, China) at room temperature for 10-15 min. After repeat washing with PBS, the cells were collected, and seeded at a density of 1×10^6^ cells/mL with α-MEM containing 10% fetal bovine serum (Gibco, USA) and 1% penicillin/streptomycin (Gibco, USA). The cell suspension was changed every 2 days. When cell confluence reached about 90%, MSCs were detached with 0.25% trypsin–EDTA (Gibco, USA) and passaged at a ratio of 1:3. MSCs from passage 3 were used in the current study.

### 2.3 Identification of MSCs by flow cytometry

MSCs (P3) were subjected to flow cytometry to determine the purity. Adherent cells were treated with 0.25% trypsin-EDTA and resuspended in PBS. Then, cells were incubated with CD44-PE antibody, CD90-PE antibody, CD45-FITC, and HLA-DR antibody (all from Invitrogen, USA). All testing was performed on a BD FACSverse instrument (BD, Franklin Lakes, NJ, USA).

### 2.4 Multi-differentiation potential assay of MSCs

MSCs were seeded into 12-well plates at a density of 2 × 10^4^ cells/cm^2^ for 24 h. Then, MSCs were cultured with an osteogenesis-induced medium, adipogenesis-induced medium and chondrogenesis-induced medium (all from Cyagen, Guangzhou, China). After 2 weeks induction, the cells were fixed with 4% PFA (Solarbio, Beijing, China) at room temperature for 20 min, and washed twice with PBS. The osteogenesis, chondrogenesis and adipogenesis were stained using Alizarin Red S, Alcian Blue and Oil Red O staining, respectively.

### 2.5 LT model and treatment

**Figure 1:**
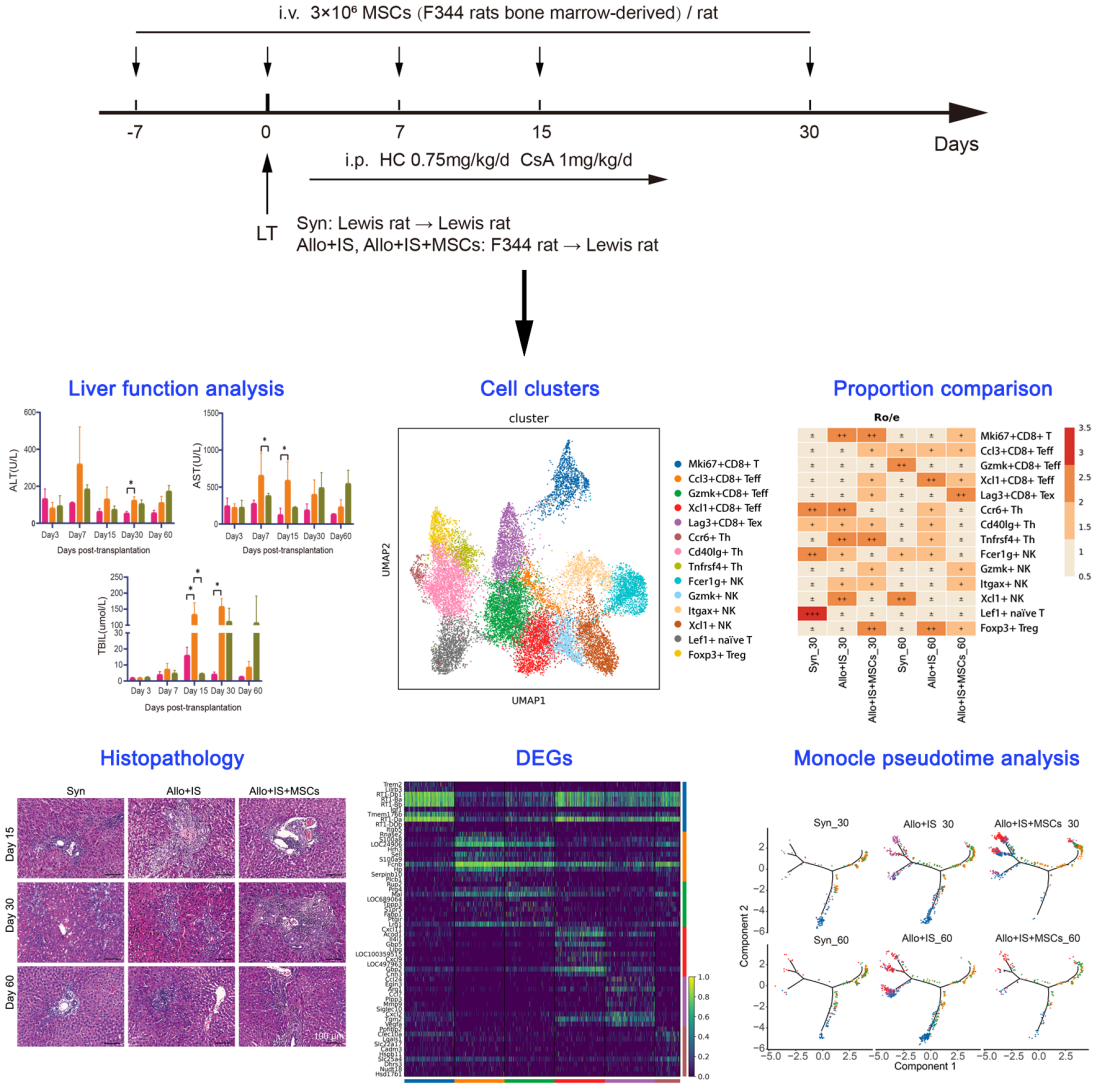
Study design schematics.

Orthotopic LT was performed with a technique described by Kamada and Calne without anastomosis of the hepatic artery (30). Rats were divided into 3 groups: 1) the syngeneic group (Syn) (n = 8), in which both the donors and recipients were Lewis rats and the recipients received saline; 2) the allogenic group (Allo+IS) (n = 8), in which the donors were F344 rats and the recipients were Lewis rats; 3) the MSC group (Allo+IS+MSCs) (n = 8), in which the donors were F344 rats and the recipients were Lewis rats. The recipients in the Allo+IS and Allo+IS+MSCs groups received intraperitoneal injection of ciclosporin A (1 mg/kg/day) and hydrocortisone (0.75 mg/kg/day) everyday starting on day 1 after LT until day 30. Freshly prepared F344 rat MSCs (3 × 10^6^ MSC diluted in 1 mL of saline) were injected into Lewis rats in the Allo+IS+MSCs group via the vena dorsalis penis 7 days before LT, and on day 0, day 7, day 15, day 30 after LT, according to a previous method (31). The study design schematics were shown in **Figure 1**.

### 2.6 Blood biochemistry

Peripheral blood from each animal was sampled from a vena at 3, 7, 15, 30 and 60 days after LT. Blood samples were centrifuged at 3,000 × g at 4°C for 10 min. Alanine aminotransferase (ALT), aspartate aminotransferase (AST) and total bilirubin (TBIL) levels were measured using an automatic analyzer.

### 2.7 Treg cell proportions using flow cytometry

Peripheral blood was collected 7, 15, and 30 days after LT. Peripheral blood mononuclear cells (PBMCs) were separated by density gradient equilibrium centrifugation using Ficoll-Hypaque (Solarbio, Beijing, China). Then, PBMCs (1×10^6^) were stained with CD4-FITC antibody (Santa cruz, USA) and CD25-Alexa Fluor 647 antibody (Bio-Rad, USA). Afterwards, PBMCs were fixed and permeabilized with intracellular staining permeabilization wash buffer (Thermo, USA) and incubated with Foxp3-PE antibody (Thermo, UAS) for 30 min at room temperature in the dark. After washing procedure, cell pellets were resuspended in staining buffer and analyzed by BD FACSverse instrument.

### 2.8 Histological examination

Liver tissues were collected 15, 30 and 60 days after LT. The tissues were fixed with 4% paraformaldehyde at room temperature for 24 h, embedded in paraffin, and cut into 5-µm thickness slides. The formalin-fixed and paraffin-embedded sections were stained by hematoxylin and eosin (H&E) stain according to the description of H&E kit (Solarbio) and their structure was observed under a microscope. To observe fibrotic changes in liver tissues, Masson trichrome staining was performed using a commercial kit (Solarbio) according to the manufacturer’s protocol. The sections were stained by Masson’s trichrome to evaluate stage fibrosis.

### 2.9 Immunofluorescence analysis

Liver tissues were cut into 5-µm thickness slides and incubated with anti-CD68 (Servicebio) and anti-CD163 (Servicebio) overnight at 4°C, followed by incubation with secondary Cy3-labelled antibody (Servicebio) and HRP-labelled antibody (Servicebio) for 50 min at room temperature. Nuclei were counterstained with DAPI.

### 2.10 Quantitative real-time PCR

Liver tissues were collected 15 days after LT. Total RNA was extracted from the recipient’s liver using TRIzol reagent (TransGen, Beijing, China). RNA extract was reversely transcribed into cDNA with the Reverse Transcript Reagents kit (Yeasen, Shanghai, China). The analysis was performed in StepOnePlusTM Real-Time PCR machine (Applied Biosystems). All samples were normalized according to 18s rRNA expression. The primer sequences were shown in **Table 1**. The results were statistically analyzed using the 2^-△△CT^ method.

**Table 1:** Primers used in mRNA expression analysis.

Gene name	Forward (5’-3’)	Reverse (5’-3’)
*IL-18*	ACCACTTTGGCAGACTTCACT	CTGGGATTCGTTGGCTGTTC
*NF-kB*	AGAGAAGCACAGATACCACTAAGA	GTTCAGCCTCATAGAAGCCATC
*TNF-α*	TAGCCCACGTCGTAGCAAAC	GTGAGGAGCACGTAGTCGG
*TNF-β*	TCCCAGTACCCCTTCCATGT	TGTAAGTGGGAGATGCCGTC
*IFN-γ*	GCCATCAGCAACAACATAAGTG	CGCTTCCTTAGGCTAGATTCTG
*18s rRNA*	CGCTTCCTTAGGCTAGATTCTG	AGAGTCTCGTTCGTTATCGGAAT

### 2.11 Rat liver tissue dissociation

Liver tissues were procured 30 days and 60 days after LT. The tissues were stored in GEXSCOPE™ Tissue Preservation Solution (Singleron). The tissues were washed with Hanks Balanced Salt Solution (HBSS) three times to remove the residual non-liver cells. The tissues were cut into 1-2 mm dimeter species. Single-cell isolation was carried out in GEXSCOPE™ tissue dissociation solution (Singleron). The dissociated single cells were collected by filtering using a 40 μm strainer. The erythrocyte lysis step proceeded with GEXCOPE™ erythrocyte lysate (Singleron).

### 2.12 Single-cell transcriptome by RNA-seq

The cell samples were re-suspended in PBS. Trypan blue staining was used to determine cell viability. Before loading onto the 10× Genomics Single-Cell-A Chip, the cell concentration was adjusted to 1.5×10^5^-5.0×10^5^ cells/mL. GEXSCOPE™ Single Cell RNA Library Kit Tissue (Singleron) was used to barcode single cells, capture mRNA from isolated single cells, and generate cDNA libraries for scRNA-seq. The sample was diluted to 4 ng/μl and sequenced on an Illumina HiSeq X sequencing platform using the 150-bp double-end mode (Illumina).

### 2.13 Read processing of scRNA-seq data

Raw sequencing data were processed to generate gene expression profiles using CeleScope v1.5.2 (Singleron) with default parameters. In brief, barcodes and unique molecular identifiers (UMI) were extracted from read 1 and corrected. Adapter sequences and poly A tails were trimmed from R2 reads. The trimmed R2 reads were aligned to the GRCh38 (hg38) transcriptome using STAR(v2.6.1b). Uniquely mapped reads were then assigned to exons with FeatureCounts(v2.0.1). Successfully assigned reads with the same cell barcode, UMI and gene were grouped together to generate the gene expression matrix for further analysis.

### 2.14 Cell filtering, dimension-reduction and clustering

The R package Seurat v 3.1.2 was used for cell filtering, dimensionality reduction and clustering. For each sample dataset, we filtered expression matrix by the following criteria: 1) cells with gene count less than 200 or with top 2% gene count were excluded; 2) cells with top 2% UMI count were excluded; 3) cells with mitochondrial content > 20% were excluded; 4) genes expressed in less than 5 cells were excluded. Gene expression matrix was normalized and scaled using the functions NormalizeData and ScaleData. The top 2,000 variable genes were selected by FindVariableFeatures for PCA. Cells were separated into 20 clusters by FindClusters, using the top 20 principal components and resolution parameter at 0.5. Cell clusters were visualized using Uniform Manifold Approximation and Projection (UMAP) with Seurat function RunUMAP and t-distributed stochastic neighbor embedding (tSNE) with RunTSNE.

### 2.15 Analysis of differentially expressed genes

DEGs were identified by the Seurat FindMarkers function based on Wilcoxon rank sum test with default parameters. The genes expressed in more than 10% of the cells in both of the compared groups of cells and with an average log(Fold Change) value of greater than 0.25 were selected as DEGs. Adjusted p value was calculated by Bonferroni Correction and the value 0.05 was used as the criterion to evaluate the statistical significance.

### 2.16 Cell type annotation

The cell type identification of each cluster was determined according to the expression of canonical markers from the reference database SynEcoSys™ (Singleron Biotechnology). SynEcoSys™ contains collections of canonical cell type markers for single-cell seq data, from CellMakerDB, PanglaoDB and recently published literatures.

### 2.17 Pathway enrichment analysis

Kyoto Encyclopedia of Genes and Genomes (KEGG) analysis was used to predict the biological function, cellular composition, and possible pathways involved in DEGs, with an adjusted P-value of less than 0.05 being considered statistically significant.

### 2.18 Pseudotime trajectory analysis: monocle2

Cell differentiation trajectory of monocyte subtypes was reconstructed with the Monocle2 v 2.10.0 (ref). For constructing the trajectory, top 2000 highly variable genes were selected by Seurat(v3.1.2) FindVairableFeatures(), and dimension-reduction was performed by DDRTree(). The trajectory was visualized by plot_cell_trajectory() function in Monocle2.

### 2.19 Trajectory switch gene analysis

To discover the order of gene expression and the function during cell state transitions, switch gene analysis was performed by using GeneSwitches (V0.1.0) in R version 3.6.3. Genes with a distinct bimodal “on-off” distribution were logistically regressed to pseudotime and the switching point was estimated as the time point when the fitted line crossed the probability threshold 0.5. Top switch genes with high McFadden’s Pseudo R^2 were plotted by plot_timeline_ggplot(). To better understand the function of switch genes, a pathway analysis was applied by using find_switch_pathway(), with GO, KEGG and MSigDB hallmark pathways included. To remove redundant pathways, the function reduce_pathways() was used with rate fixed at 0.8. Top significantly changed pathways were plotted and ordered by the swiching time using plot_pathway_density(). To compare switch genes from two trajectories, common switching genes were identified and visualized by function cmmon_genes() and common_genes_plot(), while distinct switching genes were identified and visualized by distinct_genes() and plot_timeline_ggplot().

### 2.20 Discovering the DEGs along with the trajectory

TradeSeq (v1.6.0) was used to discover the DEGs along with the trajectory. The fitGAM function in tradeSeq package was used to model the association between pseudotime and gene expression along each branch. Wald test method was applied to determine the differential expression of genes.

### 2.21 Statistical analysis

Data were expressed as the mean ± standard deviation (SD). All the statistical analyses were performed with GraphPad Prism (version 8.0). T test was used to assess the statistical analysis between the two groups. p < 0.05 was considered as statistically significant.

## 3 Results

### 3.1 Identification of isolated MSCs and effects of MSCs on liver function

**Figure 2:**
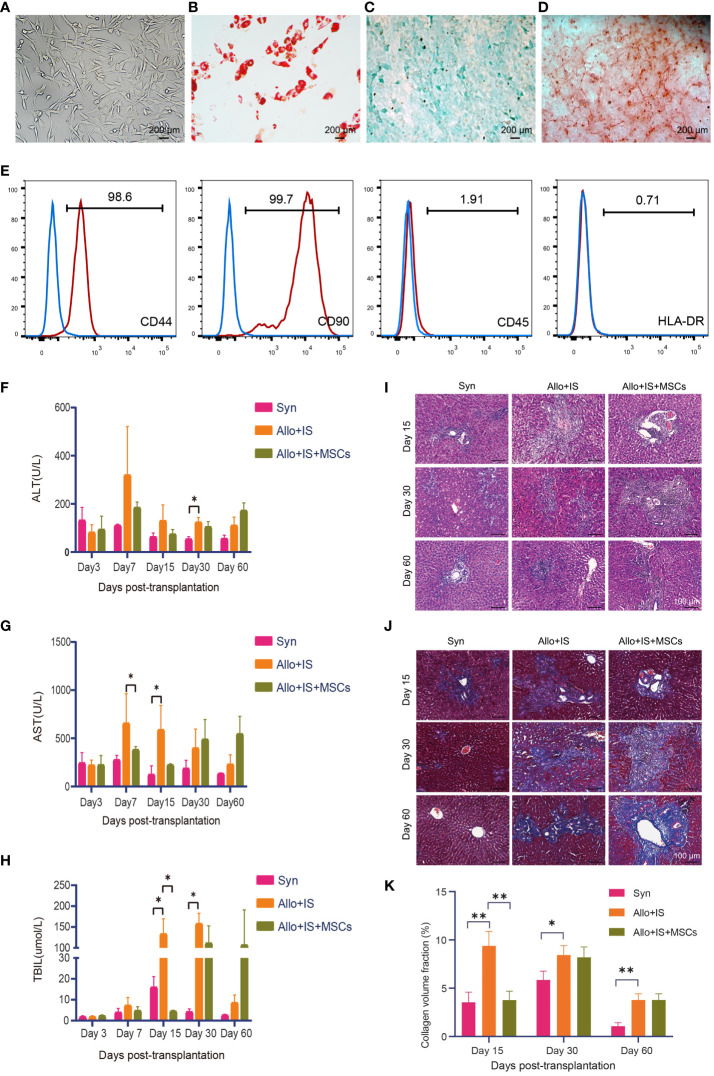
Phenotypic features of F344-derived MSCs and effects of MSCs on liver functions in rats after LT. **(A)** Morphologic features of rodent BM-derived MSCs (200 μm). **(B)** Oil red O staining of adipogenesis derived from MSCs (200 μm), **(C)** Alcian blue staining of chondrogenesis, and **(D)** Alizarin red S staining of osteoblasts. **(E)** Immunophenotyping of *in vitro* expanded and BM-derived MSCs from F344-derived MSCs; MSCs were stained with FITC- or PE-conjugated antibodies. Blood biochemistry analysis for ALT **(F)**, AST **(G)**, and TBIL **(H)**; *p < 0.05; n = 3 for each group. Microscopic images of **(I)** H&E staining (100 μm) and **(J)** Masson’s trichrome staining (100 μm) from the liver tissues. **(K)** The collagen volume fraction in liver tissues by Masson’s trichrome staining; *p < 0.05, **p < 0.01; n = 3 for each group. MSCs, mesenchymal stem cells; LT, liver transplantation; BM, bone marrow; FITC, fluorescein isothiocyanate; PE, phycoerythrin; ALT, alkaline transaminase; AST, aspartate transaminase; TBIL, total bilirubin; H&E, hematoxylin eosin; Syn, syngeneic group; Allo, allogenic group; IS, immunosuppressant.

F344 rats BM-derived MSCs were successfully expanded, and the expanded MSCs displayed large flat cells and spindle-shaped cells, which were typical morphologic features for MSCs (**Figure 2A**). Culture-expanded MSCs were able to differentiate into the adipogenic, chondrogenic and osteogenic lineages, which were specified by Oil red O (**Figure 2B**), Alcian blue (**Figure 2C**), and Alizarin red S staining (**Figure 2D**), respectively. Flow cytometry was performed to investigate the immunophenotypic characteristics of BM-MSCs. The expanded MSCs were positive for hematopoietic cell surface marker (i.e. CD44) and mesenchymal cell surface marker (i.e. CD90), while were negative for CD45 and HLA-DR (**Figure 2E**), which complied with the International Society for Cell and Gene Therapy (ISCT)-established minimal criteria to define MSCs identity. Rats in each group were monitored continuously for 100 days. There were no deaths in the Syn group. In the Allo+IS group, there was one death on day 7 and another on day 9 postoperatively. In the Allo+IS+MSCs group, there was one death on day 10 and another on day 62 postoperatively, until sacrifice. The Allo+IS group had significantly higher ALT 30 days post LT than the Syn group (**Figure 2F**).

Compared to the Allo+IS group, Allo+IS+MSCs group showed decreased AST level 7 days post LT (**Figure 2G**). At 15 days post LT, AST level increased in the Allo+IS group compared to the Syn group (**Figure 2G**). The Allo+IS group showed increased TBIL level compared to the Syn group 15 days and 30 days post LT (p < 0.05), while the Allo+IS+MSCs group had decreased TBIL compared to the Allo+IS group 15 days post LT (p < 0.05) (**Figure 2H**). Histological examination revealed pathological features in the Syn, Allo+IS, and Allo+IS+MSCs groups 15, 30 and 60 days post LT. In the Syn group, immune cell infiltration was observed in the portal tracts 15 days post LT (**Figure 2I**). Both the Allo+IS and Allo+IS+MSCs groups showed bile duct hyperplasia accompanied by degenerative changes and inflammatory cell infiltration involving the portal ducts 15 days post LT. Besides, there were enlargement of portal ducts and tissue edema in the Allo+IS group. After 30 days of LT, all three groups showed bile duct hyperplasia with degenerative changes and inflammatory cell infiltration involving most of the portal ducts. The Allo+IS group was observed with hepatic congestion and partial necrosis of hepatocytes. The Allo+IS+MSCs exhibited enlarged portal ducts. After 60 days of LT, the Syn group displayed inflammatory cells infiltrating part of the portal ducts; in the Allo+IS group, bile duct hyperplasia with degenerative bile duct changes was observed, inflammatory cell infiltration involved most of the portal ducts, and portal ducts were enlarged; in the Allo+IS+MSCs group, inflammatory cells infiltrating involved part of the portal ducts.

Masson’s trichrome staining confirmed the presence of cirrhosis in the Allo+IS group that was more severe than the Syn group or the Allo+IS+MSCs group (**Figure 2J**). The collagen volume fraction in the liver was then quantified using Image J. **Figure 2K** showed that compared with the Syn group, the Allo+IS group exhibited increased fraction of collagen volume 15 days, 30 days and 60 days after LT (p < 0.05, p < 0.01); compared with the Allo+IS group, the Allo+IS+MSCs group had significantly decreased collagen volume fraction 15 days after LT (p < 0.01).

### 3.2 MSCs-induced inflammatory responses after LT

**Figure 3:**
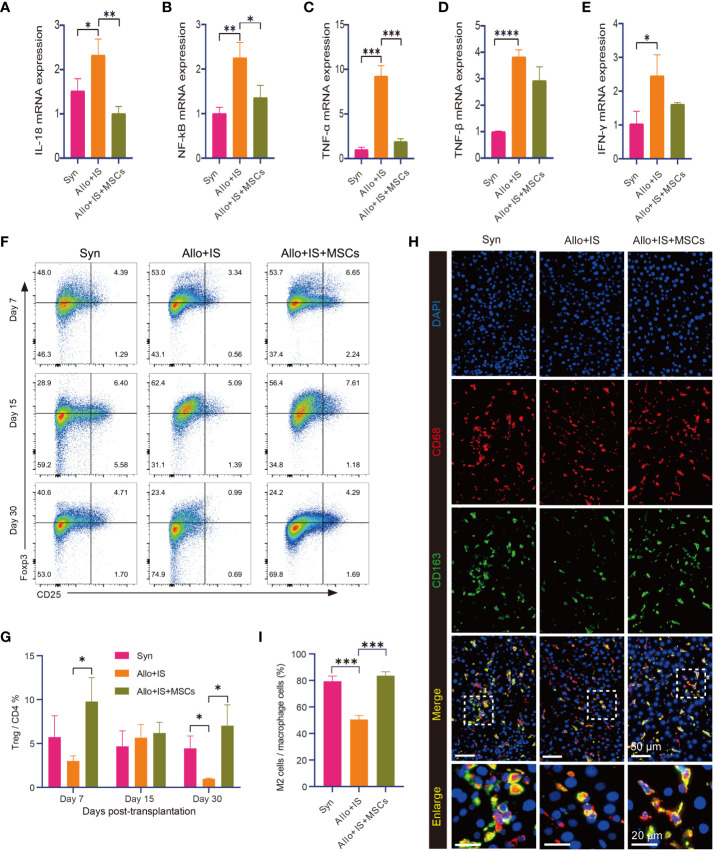
MSCs-induced immunological effects in rats after LT. qRT-PCR analysis for *IL-18* **(A)**, *NF-κB* **(B)**, *TNF-α* **(C)**, *TNF-β* **(D)**, and *IFN-γ* **(E)** 15 days post LT; *p < 0.05, **p < 0.01, ***p < 0.001, ****p < 0.0001; n = 3 for each group. **(F)** Representative dot plots of the flow cytometry analysis suggesting the distribution of Foxp3- and CD25-positive cells. **(G)** Tregs ratio in peripheral blood samples; *p < 0.05; n = 3 for each group. **(H)** Immunofluorescence staining for CD163-positive and CD68-positive macrophages (50 μm and 20 μm); DAPI for nuclear staining. **(I)** M2 macrophages (CD68+CD163+ cells) in liver tissues after LT; ***p < 0.001; n = 3 for each group. MSCs, mesenchymal stem cells; LT, liver transplantation; IL-18, interleukin-18; NF-κB, nuclear factor-kappa B; TNF, tumor necrosis factor; IFN-γ, interferon-gamma; DAPI, 4’,6-diamidino-2-phenylindole; Syn, syngeneic group; Allo, allogenic group; IS, immunosuppressant.

Notably, the Allo+IS group tended to have higher mRNA expression of *IL-18* (p < 0.05) (**Figure 3A**), *NF-κB* (p < 0.01) (**Figure 3B**), *TNF-α* (p < 0.001) (**Figure 3C**), *TNF-β* (p < 0.0001) (**Figure 3D**), and *IFN-γ* (p < 0.05) (**Figure 3E**) compared with the Syn group. In contrast, *IL-18*, *NF-κB*, and *TNF-α* were decreased in the Allo+IS+MSCs group by the comparison with the Allo+IS group (p < 0.05, p < 0.01, p < 0.001). Flow cytometry was used to identify Tregs by the expression of CD4 and CD25, and intracellular Foxp3. **Figure 3F** showed the representative dot plots of the flow cytometry analysis of Tregs from peripheral blood in the three groups 7, 15 and 30 days post LT. The Tregs ratios in the Allo+IS group was significantly lower than the ratios in the Syn group 30 days post LT (p < 0.05), while the Allo+IS+MSCs group showed an increased Treg ratio compared to the Allo+IS group 7 days and 30 days post LT (p < 0.05) (**Figure 3G**). Double immunofluorescence staining was performed with CD68 and CD163. We observed that the proportion of M2 macrophages that were co-localized with CD68-positive and CD163-positive cells, was decreased in the Allo+IS group compared to the Syn group (p < 0.001); however, the injection of MSCs enhanced its proportion when compared to the Allo+IS group (p < 0.001) (**Figures 3H, I**).

### 3.3 The landscape of intrahepatic cells in transplanted livers with the intervention of immunosuppressant and MSCs

**Figure 4:**
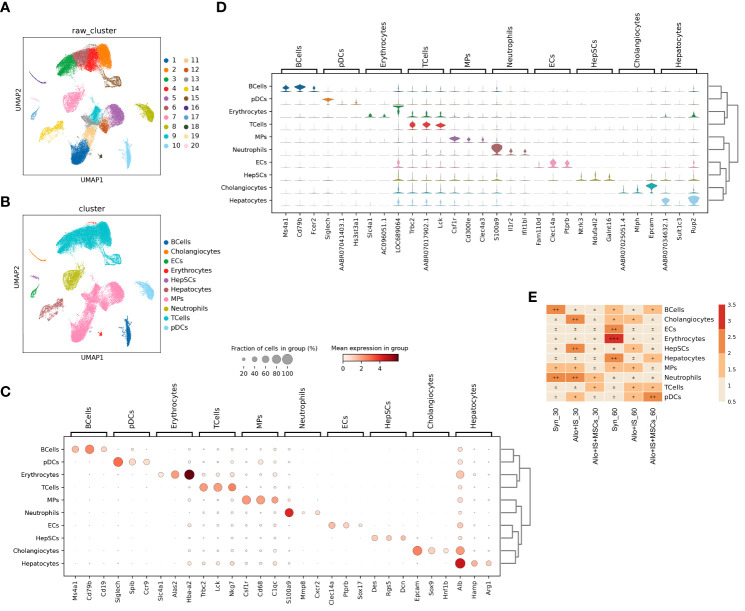
Single-cell RNA sequencing profiling of intrahepatic cells from pooled liver samples. **(A)** UMAP displaying 20 distinct cell populations in rat liver through unsupervised clustering. **(B)** Integrated UMAP showing the 10 clusters classified in rat liver. **(C)** Dot plots suggesting the top 3 highly expressed marker genes (x-axis) of 10 predominant cell types (y-axis); The size of the dot indicates the fraction of cell types and the color of the dots is the expression level of the marker genes. **(D)** Violin plots showing the top 3 DEGs (x-axis) between different cell types (y-axis). **(E)** Heatmap suggesting the proportion of 10 cell types in each sample. UMAP, uniform manifold approximation and projection; DEGs, differentially expressed genes.

To characterize the landscape of cell subpopulations within the allograft after the intervention of immunosuppressant and MSCs, we initially performed an integrated analysis of cell-type identification using the pooled samples. Single-cell sequence of intrahepatic cells obtained 53,192 single-cell transcriptomes after quality control filtering. Clustering of the intrahepatic cells obtained 20 subpopulations as visualized by UMAP (**Figure 4A**). The 20 clusters were annotated using canonical marker genes, across 10 major cell lineages (**Figure 4B**), including B cells (*Ms4a1*, *Cd79b*, *Cd19*) (32), cholangiocytes (*Epcam*, *Sox9*, *Hnf1b*) (33), endothelial cells (ECs) (*Clec14a*, *Ptprb*, *Sox17*) (34), erythrocytes (*Slc4a1*, *Alas2*, *Hba-a2*) (35), hepatic stellate cells (hepSCs) (*Des*, *Rgs5*, *Dcn*) (36), hepatocytes (*Alb*, *Hamp*, *Arg1*) (37), mononuclear phagocytes (MPs) (*Csf1r*, *Cd68*, *C1qc*) (38), neutrophils (*S100a9*, *Mmp8*, *Cxcr2*) (32), T cells (*Trbc2*, *Lck*, *Nkg7*) (39), and plasmacytoid dendritic cells (pDCs) (*Siglech*, *Spib*, *Ccr9*) (40) (**Figure 4C**). The differentially gene expression analysis showed the top 10 DEGs between different clusters, suggesting that each cluster had a characteristic gene signature (**Additional Figure 1**). **Figure 4D** listed the top 3 highly expressed DEGs, for example, *Ms4a1*, *Cd79b*, and *Fcer2* in B cells.

The distribution of the 10 cell types in each sample was shown in **Figure 4E**. In the Syn group, the proportions of cholangiocytes, ECs, erythrocytes, hepatocytes, and pDCs increased 60 days post LT compared to 30 days post LT. In contrast, proportions of B cells, T cells, and neutrophils decreased. In the Allo+IS group, the proportions of cholangiocytes, HepSCs, and neutrophils decreased, while T cells increased 60 days post LT compared to 30 days post LT. In the Allo+IS+MSCs group, the proportions of B cells, hepatocytes, and pDCs enhanced, while neutrophils decreased. As for 60 days after LT, the proportions of B cells, ECs, erythrocytes, and hepatocytes decreased, while HepSCs, T cells and pDCs increased in the Allo+IS group compared to the Syn group. In the Allo+IS+MSCs group, B cells, hepatocytes, and pDCs increased, while cholangiocytes, HepSCs, and MPs decreased compared to the Allo+IS group.

This finding implied that the application of immunosuppressant particularly in combination with MSCs altered the landscape of intrahepatic cells in transplanted livers. Specifically, the application of MSCs protected rat liver against damages after LT and inhibited inflammation to a certain degree. The hepatocytes and the corresponding markers (*AABR07034632.1*, *Sult1c3*, *Rup2*) can be used to indicate the damage of liver tissue. The immune response may be indicated by the proportion of B cells and neutrophils, as well as their marker genes (*Ms4a1* and *Cd79b* for B cells; *S100a9*, *Il1r2*, and *Ifit1bl* for neutrophils). To further investigate the unique subtype of intrahepatic cells, we clustered and identified the specific cell phenotypes in liver tissues.

### 3.4 T cell subtypes, DEGs and monocle pseudotime analysis

#### 3.4.1 T cell subtypes

**Figure 5:**
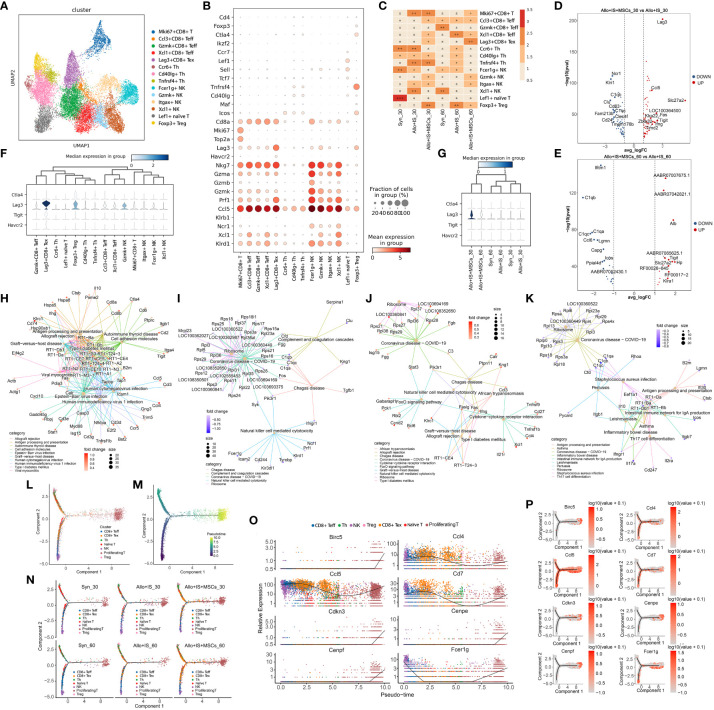
Identifying T cell subtypes in liver tissues, DEGs analysis, and monocle trajectory analysis in each sample. **(A)** UMAP plots showing the distribution of 14 T cells subtypes. **(B)** Dot plots suggesting the top 3 highly expressed marker genes (y-axis) of 14 predominant T cell subtypes (x-axis); The size of the dot indicates the fraction of T cell subtypes and the color of the dots is the expression level of the marker genes. **(C)** Heatmap presenting the proportion of 14 T cell subtypes in each sample. Volcano plots showing the top 10 DEGs between the Allo+IS and Allo+IS+MSCs 30 days **(D)** or 60 days **(E)** after LT. **(F)** Expression distribution of immune checkpoint genes (*Ctla4*, *Lag3*, *Tigit*, and *Havcr2*) in 14 T cell subtypes. **(G)** Expression distribution of immune checkpoint genes (*Ctla4*, *Lag3*, *Tigit*, and *Havcr2*) in each sample. Net plots showed the functional analysis based on the up-regulated genes **(H)** or down-regulated genes **(I)** of T cells between the Allo+IS+MSCs and Allo+IS 30 days after LT, and up-regulated genes **(J)** or down-regulated genes **(K)** between the Allo+IS+MSCs and Allo+IS 60 days after LT. **(L)** Monocle trajectory inference places 7 T cell clusters at discrete nodes. **(M)** Monocle pseudotime inference traces a path from the proliferating T cell cluster node to the NK and Treg cell cluster node. **(N)** Monocle trajectory inference places 7 T cell clusters at discrete nodes in each sample. **(O)** Expression distribution of top 8 genes in 7 T cell clusters. **(P)** DD tree showing the expression of top 8 genes in monocle reduction. DEGs, differentially expressed genes; UMAP, uniform manifold approximation and projection; Allo, allogenic group; IS, immunosuppressant; MSCs, mesenchymal stem cells; LT, liver transplantation; NK, natural killer cells.

There were 20,042 single cells detected in T cells, which were clustered into 14 T cell subtypes shown in UMAP (**Figure 5A**). The identification of T cell subtypes was performed according to previous method based on the highly expressed marker genes, including CD8+ effector T cells (Teff) (Gzmk+CD8+ Teff, Ccl3+CD8+ Teff, Xcl1+CD8+ Teff) (41), exhausted T cells (Tex) (Lag3+CD8+ Tex) (42), helper T cells (Th) (Cd401g+ Th, Tnfrsf4+ Th, Ccr6+ Th) (32), natural killer T cells (NKT) (Fcer1g+ NK, Itgax+ NK, Xcl1+ NK, Gzmk+ NK) (43), naïve T cells (Lef1+ naïve T cells) (44), regulatory T cells (Treg) (Foxp3+ Treg) (45), and Mki67+CD8+ T cells (**Figure 5B**). The composition of the 14 T cell subtypes was significantly altered 30 days or 60 days after LT in spite of application of immunosuppressant or MSCs (**Figure 5C**).

In the Syn group, the proportions of Ccl3+CD8+ Teff, Gzmk+CD8+ Teff, and Xcl1+ NK increased while Ccr6+ Th, Cd40lg+ Th, Fer1g+ NK, and Lef1+ naïve T cells decreased 60 days after LT compared to 30 days after LT. In the Allo+IS group, the proportions of Ccl3+CD8+ Teff, Xcl1+CD8+ Teff and Foxp3+ Treg increased, whereas we noted decreased proportions of Mki67+CD8+ T cells, Ccr6+ Th, Tnfrsf4+ Th, Itgax+ NK and Xcl1+ NK 60 days after LT relative to 30 days after LT. As for the Allo+IS+MSCs group, the proportions of Mki67+CD8+ T cells, Cd40lg+ Th, Tnfrsf4+ Th, and Foxp3+ Treg reduced whereas Lag3+CD8+ Tex increased over time. Compared to the Syn group, the Allo+IS group demonstrated marked increases in the proportions of Mki67+CD8+ T cells, Tnfrsf4+ Th, Itgax+ NK and Xcl1+ NK, while decreases in Fcer1g+ NK and Lef1+ naïve T cells 30 days after LT; At 60 days after LT, Xcl1+CD8+ Teff, Ccr6+ Th, Cd40lg+ Th, Tnfrsf4+ Th, and Foxp3+ Treg increased in Allo+IS group compared with those of Syn group, while Gzmk+CD8+ Teff and Xcl1+ NK decreased. At 30 days after LT, the Allo+IS+MSCs group showed increased proportions of Ccl3+CD8+ Teff, Xcl1+CD8+ Teff, Lag3+CD8+ Tex, Gzmk+ NK and Foxp3+ Treg, while decreased proportions of Ccr6+ Th, Fcer1g+ NK and Xcl1+ NK when compared to the Allo+IS group. At 60 days after LT, the proportions of Mki67+CD8+ T cells, Lag3+CD8+ Tex, Gzmk+ NK and Itgax+ NK increased, and the proportions of Xcl1+CD8+ Teff, Ccr6+ Th, Cd40lg+ Th, Tnfrsf4+ Th, Fcer1g+ NK and Foxp3+ Treg decreased in the Allo+IS+MSCs group relative to the Allo+IS group.

#### 3.4.2 DEGs in T cell subtypes among the samples

**Additional File 1** showed the DEGs of T cells in the Syn group 30 days after LT compared to the 60 days after LT. The DEGs were compared between 30 days after LT and 60 days after LT, for the Allo+IS or Allo+IS+MSCs, which were presented in **Additional Files 2** and **3**. We next analyzed the DEGs between the Allo+IS group and Syn group 30 days (**Additional File 4**) and 60 days (**Additional File 5**) after LT. At 30 days after LT, the top 10 genes highly enriched in the Allo+IS+MSCs group compared to the Allo+IS group included *Lag3*, *Ccl5*, *Sl27a2*, *LOC100364500*, *Fas*, *Klra22*, *Tigit*, *Zbtb32*, *Ifng*, and *Timd2*, and the down-regulated genes were *Ncr1*, *Klri1*, *C1qb*, *Clu*, *Cd63*, *C1qc*, *Fam213b*, *Clec4f*, *Cd24*, and *Tmem176b* (**Figure 5D**). At 60 days after LT, the top 10 genes highly enriched in the Allo+IS+MSCs group compared to the Allo+IS group included *AABR07007675.1*, *AABR07042821.1*, *Alb, AABR07065625.1*, *Tigit*, *Slc27a2*, *Hp*, *RF00026-845*, *RF00017-2*, and *Klra1*, and the down-regulated genes were *Ifitm1*, *C1qb*, *C1qa*, *C1qc*, *Ccl6*, *Lgmn*, *Capg*, *Icos*, *Ppial4d*, and *AABR07052430.1* (**Figure 5E**). **Figure 5F** showed the distribution of immune checkpoint genes (*Ctla4*, *Lag3*, *Tigit*, and *Havcr2*) in 14 T cell subtypes. Of the 4 immune checkpoint genes, *Lag3* gene was identified as the feature gene of Lag3+CD8+ Tex. The expression distribution of the immune checkpoint genes in each sample was shown in **Figure 5G**. Particularly, *Lag3* gene was highly expressed in the Allo+IS+MSCs group 30 days after LT compared to the Allo+IS group. Functional analysis revealed that these up-regulated DEGs may be implicated in the regulation of allograft rejection and antigen processing and presentation (**Figure 5H**), and down-regulated genes may mediate complement and coagulation cascades (**Figure 5I**) 30 days after LT. As for 60 days after LT, the up-regulated genes may be related to allograft rejection (**Figure 5J**), and the down-regulated genes may mediate antigen processing and presentation (**Figure 5K**).

#### 3.4.3 Monocle pseudotime analysis revealed potential paths of T cell differentiation

Then trajectory analysis was performed to indicate the transitional states of pooled T cells from all samples. Based on the DEGs, T cells were clustered into 7 clusters, including CD8+ Teff (Ccl3+CD8+ Teff, Gzmk+CD8+ Teff, Xcl1+CD8+ Teff), CD8+ Tex (Lag3+CD8+ Tex), Th (Ccr6+ Th, Cd401g+ Th, Tnfrsf4+ Th), naïve T (Lef1+ naïve T cells), NK (Fcer1g+ NK, Itgax+ NK, Xcl1+ NK, Gzmk+ NK), Treg (Foxp3+ Treg), and proliferating T cells (Mki67+CD8+ T cells). Three T cell clusters (proliferating T cells, Treg, and NK) were clustered at discrete nodes (**Figure 5L**). CD8+ Teff, CD8+ Tex, Helper T, and naïve T cells were placed in the branch, presenting their transitional states. Next, we placed T cell subpopulations along a trajectory of pseudotime, and we speculated that the trajectory began at the NK and Treg nodes and ended at the proliferating T cells (**Figure 5M**). The branch of the trajectory consisted mostly of CD8+ Teff, CD8+ Tex, Th, and naïve T cells, indicating that these T subtypes represented transitional cell states in this particular differentiation path. **Figure 5N** displayed that T cell states were significantly different between the samples. It was indicated that intra-graft T cells differentiate from CD8+ Tex, CD8+ Teff or NK cells to proliferating T cells in the context of LT with immunosuppressant and MSCs. It seems that the application of immunosuppressant inhibited the generation of proliferation T cells of the Allo rats, while in the Allo+IS+MSCs group, the differentiation of CD8+ Tex may be induced into proliferating T cells. We next analyzed the expression of *Birc5*, *Ccl4*, *Ccl5, Cd7, Cdkn3, Cenpe, Cenpf*, and *Fcer1g*. **Figure 5O** suggested that *Ccl5*, *Ccl4*, and *Cd7* were predominantly expressed by NK, CD8+ Teff, CD8+ Tex and proliferating T cells. *Birc5*, *Cdkn3*, *Cenpf*, and *Cenpe*, and *Fcer1g* were highly expressed in NK and proliferating T cells. Proliferating T cells significantly expressed all these genes. **Figure 5P** showed that *Ccl5*, *Ccl4*, *Cd7* and *Fcer1g* were expressed throughout T cell differentiation.

These results showed that immunosuppressant alone or in combination with MSCs affected the proportions of T cells after LT. Through examination of the proportion of T cells subclusters (Lag3+CD8+ Tex, Foxp3+ Treg, Gzmk+ NK) and marker genes (*Lag3*), T cell-mediated immune responses can be evaluated for patients using immunosuppressive drugs after LT.

### 3.5 MPs subtypes and DEGs analysis

#### 3.5.1 MPs subtypes

**Figure 6:**
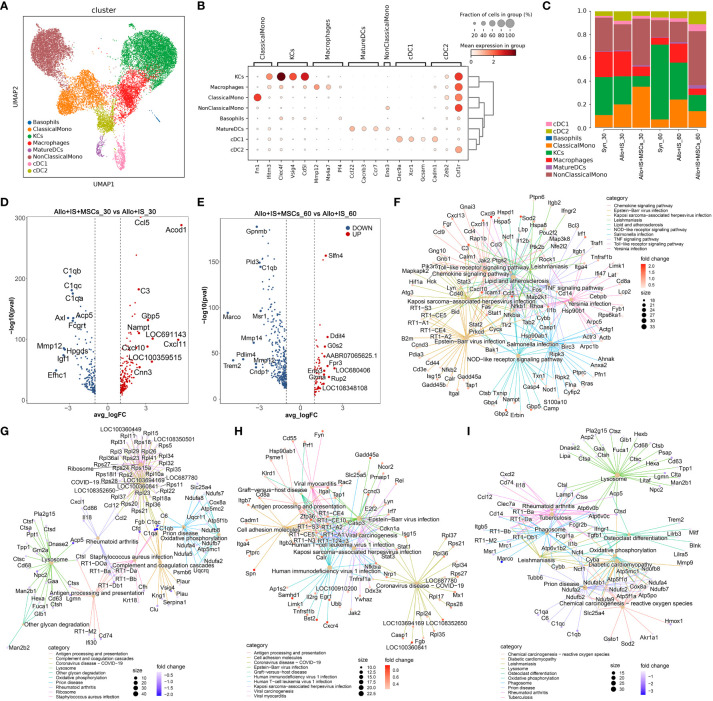
Identifying MPs subtypes in liver tissues and DEGs analysis in each sample. **(A)** UMAP plots showing the distribution of 8 MPs subtypes. **(B)** Dot plots suggesting the top 3 highly expressed marker genes (x-axis) of 8 predominant MPs subtypes (y-axis). The size of the dot indicates the fraction of subtypes and the color of the dots is the expression level of the marker genes. **(C)** Bar plots showing the proportion of 8 MPs subtypes in each sample. Volcano plots showing the top 10 differentially expressed genes between the Allo+IS and Allo+IS+MSCs 30 days **(D)** or 60 days **(E)** after LT. Net plots showed the functional analysis based on the up-regulated genes **(F)** or down-regulated genes **(G)** of MPs between the Allo+IS+MSCs and Allo+IS 30 days after LT, and up-regulated genes **(H)** or down-regulated genes **(I)** between the Allo+IS+MSCs and Allo+IS 60 days after LT. MPs, mononuclear phagocytes; DEGs, differentially expressed genes; UMAP, uniform manifold approximation and projection; Allo, allogenic group; IS, immunosuppressant; MSCs, mesenchymal stem cells; LT, liver transplantation.

MPs were clustered to 8 subtypes: basophils, classical monocytes (ClassicalMono), hepatic macrophages (traditionally called Kupffer cells, KCs), macrophages, mature dendritic cells (MatureDCs), non-classical monocytes (NonClassicalMono), conventional type 1 dendritic cells (cDC1) and cDC2 (**Figure 6A**). The identification of MPs subtypes was carried out with the highly expressed marker genes, including ClassicalMono (*Fn1*, *Ifitm3*) (32), KCs (*Clec4f*, *Vsig4*, *Cd5l*) (46), Macrophages (*Mmp12*, *Ms4a7*, *Pf4*) (47), MatureDCs (*Ccl22*, *Cacnb3*, *Ccr7*) (48), NonClassicalMono (*Eno3*) (49), cDC1 (*Clec9a*, *Xcr1*, *Gcsam*) (50), and cDC2 (*Cadm1*, *Zeb2*, *Csf1r*) (51), according to previous reports. **Figure 6B** showed the mean expression of marker genes in each cell subtype. The composition of MPs subtypes was then analyzed.

The Syn group exhibited increased proportions of KCs, MatureDCs, cDC1, and cDC2, and decreased proportions of basophils, ClassicalMono, macrophages and NonClassicalMono 60 days post LT compared to 30 days post LT; In the Allo+IS group, ClassicalMono, KCs, MatureDCs, and cDC1 increased, while basophils, macrophages, NonClassicalMono and cDC2 decreased; In the Allo+IS+MSCs group, basophils, KCs, MatureDCs, NonClassicalMono, cDC1, and cDC2 increased, and ClassicalMono and macrophages decreased (**Figure 6C**). At 30 days after LT, the proportions of ClassicalMono, MatureDCs, cDC1 and cDC2 increased, while basophils, KCs, macrophages and NonClassicalMono decreased in the Allo+IS group, compared to the Syn group; At 60 days after LT, ClassicalMono, macrophages, MatureDCs, NonClassicalMono, cDC1 and cDC2 increased, while basophils and KCs decreased in the Allo+IS group relative to the Syn group. In terms of the Allo+IS+MSCs group, the proportions of ClassicalMono, MatureDCs and NonClassicalMono increased, while basophils, KCs, macrophages, cDC1 and cDC2 decreased relative to the Allo+IS group at 30 days after LT; At 60 days after LT, the combination of immunosuppressant and MSCs increased the proportions of basophils, MatureDCs, NonClassicalMono, cDC1 and cDC2, while decreased the proportions of ClassicalMono, KCs and macrophages compared to the Allo+IS group.

#### 3.5.2 DEGs in MPs subtypes among the samples

The DEGs of MPs were shown in **Additional File 6**, when comparing 30 days after LT to 60 days after LT in the Syn group. **Additional File 7** and **8** showed the DEGs of MPs between 30 days after LT and 60 days after LT for the Allo+IS and the Allo+IS+MSCs groups, respectively. The DEGs of MPs between the Allo+IS and Syn group were also compared for the samples collected 30 days or 60 days after LT, which were shown in **Additional Files 9** and **10**. **Figure 6D** indicated that compared to the Allo+IS group, *Ccl5*, *Acod1*, *C3*, *Gbp5*, *Nampt*, *LOC691143*, *Cxcl11*, *Cxcl10*, *LOC100359515*, *Cnn3* were enriched in the Allo+IS+MSCs group 30 days after LT, and the top 10 down-regulated DEGs were *C1qb*, *C1qc*, *C1qa*, *Acp5*, *Axl*, *Fcgrt*, *Mmp12*, *Hpgds*, *Igf1*, and *Efhc1*. Compared to the Allo+IS group 60 days after LT, the top 10 up-regulated (*Slfn4*, *Ddit4*, *G0s2*, *AABR07065625.1*, *Fpr3*, *LOC680406*, *Eno3*, *Gzma*, *Rup2*, and *LOC108348108*) or down-regulated genes (*Gpnmb*, *Pld3*, *C1qb*, *Marco*, *Msr1*, *Mmp14*, *Pdlim4*, *Mmp12*, *Trem2*, and *Cndp1*) in the Allo+IS+MSCs group were marked in the volcano (**Figure 6E**). Functional analysis revealed that the DEGs between the Allo+IS and Allo+IS+MSCs may participate in mediating chemokine signaling pathway, antigen processing and presentation, cell adhesion, and lysosome et al. (**Figures 6F–I**).

Summarily, the application of immunosuppressive agents after LT altered the proportion of macrophages. The proportion of macrophages could be used to indicate LT-induced immune reaction. Particularly, its marker gene *Mmp12* was significantly down-regulated after MSCs treatment compared with the Allo+IS group.

### 3.6 Classical monocytes subclusters, DEGs and monocle pseudotime analysis

#### 3.6.1 Classical monocytes subclusters

**Figure 7:**
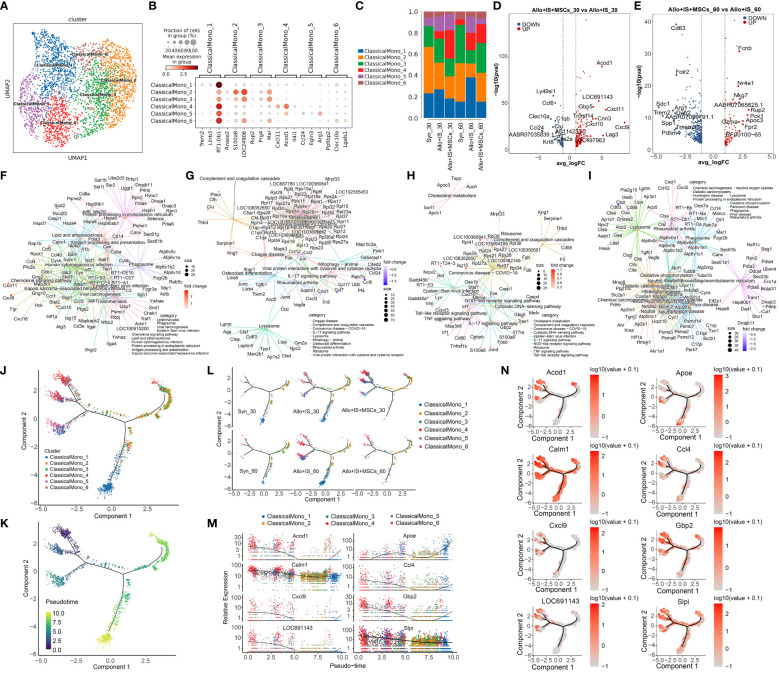
Classical monocytes subclusters, DEGs and monocle pseudotime analysis. **(A)** UMAP plots showing the distribution of 6 classical monocyte subpopulations. **(B)** Dot plots suggesting the top 3 highly expressed genes (x-axis) of 6 predominant subpopulations (y-axis); The size of the dot indicates the fraction of cell subpopulation and the color of the dots is the expression level of the marker genes. **(C)** Bar plots presenting the proportion of 6 classical monocyte subpopulations in each sample. Volcano plots showing the top 10 DEGs between the Allo+IS and Allo+IS+MSCs 30 days **(D)** or 60 days **(E)** after LT. Net plots showed the functional analysis based on the up-regulated genes **(F)** or down-regulated genes **(G)** of classical monocytes between the Allo+IS+MSCs and Allo+IS 30 days after LT, and up-regulated genes **(H)** or down-regulated genes **(I)** between the Allo+IS+MSCs and Allo+IS 60 days after LT. **(J)** Monocle trajectory inference places 6 classical monocyte cluster at discrete nodes. **(K)** Monocle pseudotime inference traces a differentiation pathway. **(L)** Monocle trajectory inference places classical monocyte clusters at discrete nodes in each sample. **(M)** Expression distribution of top 8 genes in 6 classical monocyte clusters. **(N)** DD tree showing the expression of top 8 genes in monocle reduction. UMAP, uniform manifold approximation and projection; DEGs, differentially expressed genes; Allo, allogenic group; IS, immunosuppressant; MSCs, mesenchymal stem cells; LT, liver transplantation.

Classical monocytes were grouped into 6 subpopulations, including ClassicalMono_1, ClassicalMono_2, ClasscialMono_3, ClassicalMono_4, ClassicalMono_5, and ClassicalMono_6, of which the distribution was shown in UMAP (**Figure 7A**). We analyzed the uniquely or highly expressed genes in each cluster and listed the top 10 genes of each cluster in **Additional Figure 2**. The expression of top 3 highly expressed marker genes was presented in **Figure 7B**, which was used to identify the subclusters ClassicalMono_1 (*Trem2*, *Lilrb3*, *RT1-Db1*), ClassicalMono_2 (*Rnase2*, *S100a8*, *LOC24906*), ClasscialMono_3 (*Rup2*, *Prg4*, *Mal*), ClassicalMono_4 (*Cxcl11*, *Acod1*, *Il4i1*), ClassicalMono_5 (*Ccl24*, *Egln3*, *Arg1*), and ClassicalMono_6 (*Ppfibp2*, *Clec10a*, *Lgals1*). The proportions of 6 classical monocyte subclusters was compared between the samples, which were presented in **Figure 7C**. Compared to the Allo+IS group, we observed increased proportions of ClassicalMono_4 and ClassicalMono_5 in the Allo+IS+MSCs, 30 days after LT. In contrast, the proportion of ClassicalMono_4 reduced in the Allo+IS+MSCs group compared to the Allo+IS group 60 days after LT.

#### 3.6.2 DEGs in classical monocytes subclusters

The DEGs of classical monocytes were compared between the 30 days after LT and 60 days after LT in the Syn group (**Additional File 11**). **Additional Files 12** and **13** presented the DEGs when comparing samples obtained 30 days after LT and 60 days after LT, for the Allo+IS and Allo+IS+MSCs, respectively. The DEGs between the Allo+IS and Syn group at 30 days and 60 days after LT were listed in **Additional Files 14** and **15**, respectively. We particularly pay attention to the DEGs in classical monocytes between the Allo+IS+MSCs and Allo+IS group. **Figure 7D** showed the top 10 up-regulated genes (*Acod1*, *LOC691143*, *Gbp5*, *Cxcl11*, *Cnn3*, *Tnfrsf14*, *Cxcl10*, *Cxcl9*, *LOC497963* and *Lag3*) and down-regulated genes (*Ly49si1*, *Ccl6*, *Clec10a*, *C1qb*, *Clu*, *Ccl24*, *AABR07035839.1*, *AC114233.2*, *Ctla2a* and *Krt8*) in the Allo+IS+MSCs compared to the Allo+IS 30 days after LT. For the samples collected 60 days after LT, we marked the top 10 up-regulated (*Fcnb*, *Nr4a1*, *Nkg7*, *AABR07065625.1*, *Rup2*, *Pck1*, *Apoc3*, *Gzma*, *Fpr2*, and *RF00100-65*) and down-regulated (*Cd63*, *Folr2*, *Sdc1*, *Arg1*, *Trem2*, *Dab2*, *AABR07030791.1*, *Spp1*, *Tmem37*, and *Pdlim4*) genes in the Allo+IS+MSCs compared to the Allo+IS group, which was shown in **Figure 7E**. Functional analysis revealed the potential role of these DEGs in KEGG pathway. The up-regulated genes may be included in the regulation of antigen processing and presentation, chemokine signaling pathway and complement and coagulation cascades (**Figures 7F, G**). The down-regulated genes were related to lysosome, oxidative phosphorylation, and protein processing in endoplasmic reticulum (**Figures 7H, I**).

#### 3.6.3 Monocle pseudotime analysis

Monocle pseudotime analysis revealed potential paths of classical monocyte and non-classical monocyte differentiation. We next performed the monocle trajectory inference for classical monocyte. **Figure 7J** showed that ClassicalMono_1 and ClassicalMono_4 were placed at discrete nodes, and ClassicalMono_2 and ClassicalMono_3 were placed in the right branch. It was speculated that the trajectory may began at the ClassicalMono_2 and ended at the ClassicalMono_4 and ClassicalMono_1 nodes (**Figure 7K**). **Figure 7L** suggested that ClassicalMono_1 and ClassicalMono_4 were enriched in the Allo+IS group 60 days after LT. However, the Allo+IS+MSCs group showed the opposite results. *Apoe* was highly expressed in the ClassicalMono_1, which may be considered as a marker gene for LT of the animal receiving the immunosuppressant alone (**Figure 7M**). What’s more, *Calm1* gene was expressed throughout the classical monocyte differentiation (**Figure 7N**).

### 3.7 Non-classical monocytes subclusters, DEGs and monocle pseudotime analysis

#### 3.7.1 Non-classical monocytes subclusters

**Figure 8:**
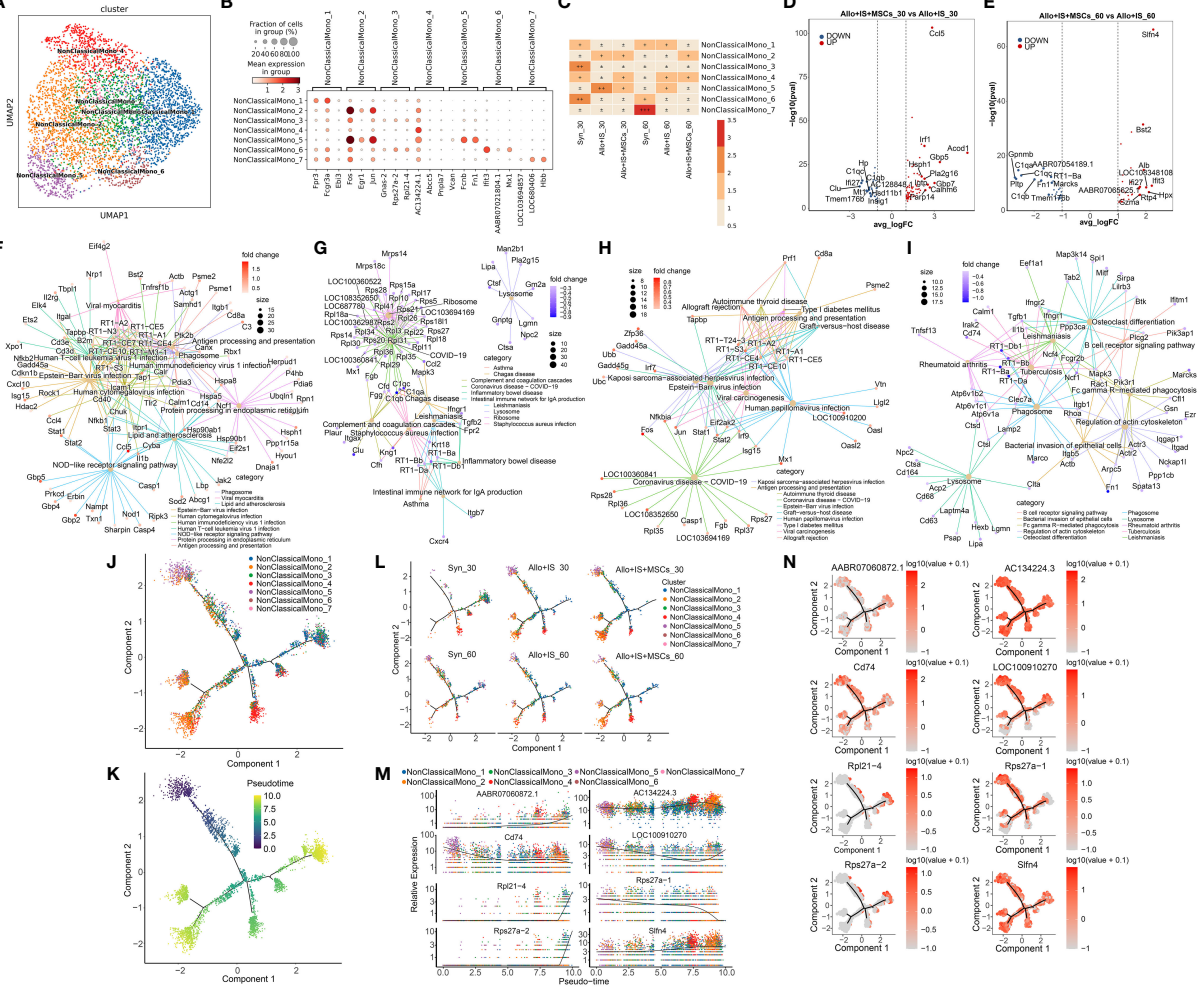
Non-classical monocytes subclusters, DEGs and monocle pseudotime analysis. **(A)** UMAP plots showing the distribution of 7 non-classical monocyte subpopulation. **(B)** Dot plots suggesting the top 3 highly expressed genes (x-axis) of 7 predominant subpopulations (y-axis); The size of the dot indicates the fraction of cell subpopulation and the color of the dots is the expression level of the marker genes. **(C)** Heatmap presenting the proportion of 7 non-classical monocyte subpopulations in each sample. Volcano plots showing the top 10 DEGs between the Allo+IS and Allo+IS+MSCs 30 days **(D)** or 60 days **(E)** after LT. Net plots showed the functional analysis based on the up-regulated genes **(F)** or down-regulated genes **(G)** of non-classical monocytes between the Allo+IS+MSCs and Allo+IS 30 days after LT, and up-regulated genes **(H)** or down-regulated genes **(I)** between the Allo+IS+MSCs and Allo+IS 60 days after LT. **(J)** Monocle trajectory inference places 7 non-classical monocyte cluster at discrete nodes. **(K)** Monocle pseudotime inference traces a differentiation pathway. **(L)** Monocle trajectory inference places classical monocyte clusters at discrete nodes in each sample. **(M)** Expression distribution of top 8 genes in 7 non-classical monocyte clusters. **(N)** DD tree showing the expression of top 8 genes in monocle reduction. UMAP, uniform manifold approximation and projection; DEGs, differentially expressed genes; Allo, allogenic group; IS, immunosuppressant; MSCs, mesenchymal stem cells; LT, liver transplantation.

Non-classical monocytes were clustered into 7 subpopulations, including NonClassicalMono_1, NonClassicalMono_2, NonClassicalMono_3, NonClassicalMono_4, NonClassicalMono_5, NonClassicalMono_6, and NonClassicalMono_7 (**Figure 8A**). The top 10 differentially or uniquely expressed genes in each subcluster were presented in the heatmap, such as *Fpr3* in NonClassicalMono_1, *Fosb* in NonClassicalMono_2, *Gnas-2* in NonClassicalMono_3, *AC134224.1* in NonClassicalMono_4, *Vcan* in NonClassicalMono_5, *Ifit3* in NonClassicalMono_6, and *LOC103694857* in NonClassicalMono_7 (**Additional Figure 3**). The top 3 genes were selected as the marker genes for each cluster, and their mean expression was illustrated in **Figure 8B**, including NonClassicalMono_1 (*Fpr3*, *Fcgr3a*, *Ebi3*), NonClassicalMono_2 (*Fos*, *Egr1*, *Jun*), NonClassicalMono_3 (*Gnas-2*, *Rps27a-2*, *Rpl21-4*), NonClassicalMono_4 (*AC134224.1*, *Abcc5*, *Pnpla7*), NonClassicalMono_5 (*Vcan*, *Fcnb*, *Fn1*), NonClassicalMono_6 (*Ifit3*, *AABR07021804.1*, *Mx1*), and NonClassicalMono_7 (*LOC103694857*, *LOC680406*, *Hbb*). Next, we compared the difference in the composition of non-classical monocyte subpopulations between the samples. **Figure 8C** showed that the proportions of non-classical monocyte subclusters were altered in the Syn, Allo+IS, and Allo+IS+MSCs when comparing the sample collected 30 days after LT to 60 days after LT. Particularly, the proportions of NonClassicalMono_3, NonClassicalMono_4 and NonClassicalMono_6 decreased 60 days after LT compared to 30 days after LT, in the Syn group. At 30 days after LT, both the Allo+IS and Allo+IS+MSCs groups showed higher proportions of NonClassicalMono_2 and NonClassicalMono_5 compared to the Syn group. Additionally, in the Allo+IS and Allo+IS+MSCs groups, we observed that NonClassicalMono_5 continually decreased at 60 days after LT compared to 30 days after LT.

#### 3.7.2 DEGs in non-classical monocytes

We performed comparison of DEGs between 30 days and 60 days after LT for the Syn, Allo+IS, and Allo+IS+MSCs, **Additional Files 16**–**18** listed the DEGs in non-classical monocytes between 30 days and 60 days after LT in the Syn, Allo+IS, and Allo+IS+MSCs, respectively. The DEGs between the Allo+IS and Syn group at 30 days and 60 days after LT were listed in Additional Files 19 and 20, respectively. Considering the difference in the composition of non-classical monocyte subpopulations between different groups, we compared the DEGs between the Allo+IS+MSCs and Allo+IS 30 days or 60 days after LT. **Figure 8D** showed the labelled top 10 genes up-regulated (*Ccl5*, *Irf1*, *Gbp5*, *Acod1*, *Hsph1*, *Pla2g16*, *Igtp*, *Gbp7*, *Calhm6*, and *Parp14*) or down-regulated (*C1qc*, *C1qb*, *AC128848.1*, *Ifi27*, *Clu*, *Hp*, *Mt1*, *Tmem176b*, *Hsd11b1*, and *Insig1*) in the Allo+IS+MSCs compared to the Allo+IS 30 days after LT. As for the sample collected at 60 days after LT, **Figure 8E** showed marked the top 10 highly expressed genes (*Slfn4*, *Bst2*, *Alb*, *Ifi27*, *Ifit3*, *AABR07065625.1*, *LOC108348108*, *Gzma*, *Rtp4*, and *Hpx*) and top 10 low expressed genes (*C1qa*, *C1qc*, *Gpnmb*, *Pltp*, *AABR07054189.1*, *C1qb*, *RT1-Ba*, *Fn1*, *Marcks*, and *Tmem176b*) in the Allo+IS+MSCs compared to the Allo+IS. Function analysis suggested their role in antigen processing and presentation, complement and coagulation cascades, allograft rejection and B cell receptor signaling pathway (**Figures 8F–I**).

#### 3.7.3 Monocle pseudotime analysis

Monocle pseudotime analysis revealed potential paths of non-classical monocyte differentiation. The results were shown in **Figure 8J**, suggesting that NonClassicalMono_4 and NonClassicalMono_5 were clearly placed at discrete nodes. We speculated that the trajectory may began at the NonClassicalMono_5 (**Figure 8K**). **Figure 8L** showed the distribution of classical monocyte clusters throughout the differentiation in each group. The proportion of the NonClassicalMono_7 was relatively low. It seems that the NonClassicalMono_7 was increased in the Syn group and the Allo+IS+MSCs group 30 days after LT compared to 60 days post LT, placed at discrete nodes. *AC134224.3*, *Cd74* and *Slfn4* genes were expressed by the 7 populations throughout the differentiation (**Figures 8M, N**).

### 3.8 KCs subclusters and DEGs

Specifically, we analyzed the DEGs between the Allo+IS and Syn or Allo+IS+MSCs and Allo+IS, 30 days and 60 days after LT. The top 10 up-regulated or down-regulated DEGs in 6 subclusters of KCs were suggested in volcano plots (**Additional Figure 4**). It was observed that *Rpl21-3*, *Rps27a-1*, *Gnas-1*, *Cox6b1-1*, and *Uqcrb-1* were down-regulated, and *Rpl21-4*, *Gnas-2*, *Rps27a-2*, and *Rps4x-2* in all KCs subclusters were up-regulated in the Syn group compared to the Allo+IS group 30 days after LT. Compared to the Allo+IS group, only *C3* in all KCs subclusters was down-regulated in the Allo+IS+MSCs group 30 days after LT. *RT1-Ba*, *RT1-Bb*, *RT1-Da*, *RT1-Db1*, and *Cd74* of all KCs subclusters were decreasingly expressed, and *Ifi27l2b* and *Fabp1* were increasingly expressed in the Syn group compared to the Allo+IS group 60 days after LT. Compared to the Allo+IS group, *Marco* of all KCs subclusters was increased in the Allo+IS+MSCs group. Summarily, the differentially expressed genes (for instance, *Rpl21-3*) by KCs subclusters can be used as immune response markers for patients receiving LT and immunosuppressive therapy.

### 3.9 pDCs subpopulations in liver tissues and comparison of DEGs in each sample

#### 3.9.1 pDCs subpopulations

**Figure 9:**
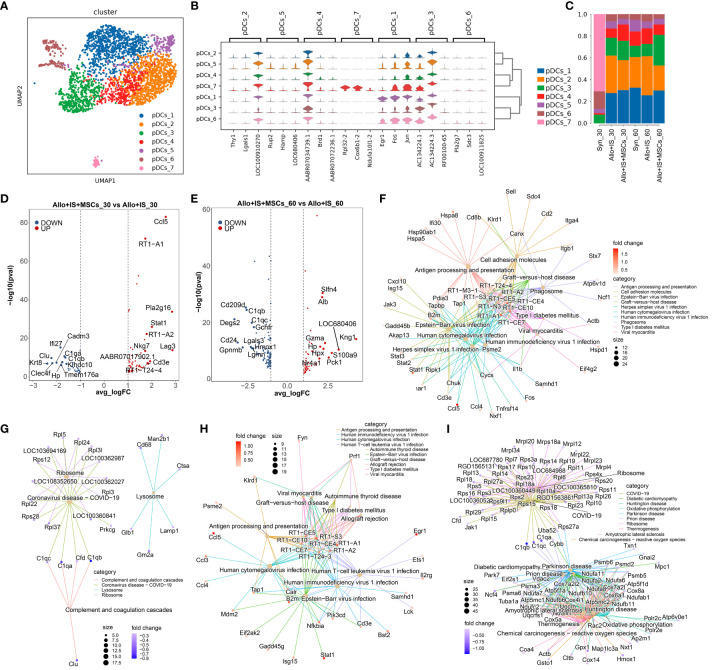
Identifying pDCs subclusters and analysis of DEGs. **(A)** UMAP plots showing the distribution of 7 pDCs subpopulations. **(B)** Dot plots suggesting the top 3 highly expressed genes (x-axis) of 7 predominant subpopulations (y-axis); The size of the dot indicates the fraction of cell subpopulation and the color of the dots is the expression level of the marker genes. **(C)** Bar plots presenting the proportion of 7 pDCs subpopulations in each sample. Volcano plots showing the top 10 differentially expressed genes between the Allo+IS and Allo+IS+MSCs 30 days **(D)** or 60 days **(E)** after LT. Net plots showed the functional analysis based on the up-regulated genes **(F)** or down-regulated genes **(G)** of pDCs between the Allo+IS+MSCs and Allo+IS 30 days after LT, and up-regulated genes **(H)** or down-regulated genes **(I)** between the Allo+IS+MSCs and Allo+IS 60 days after LT. pDCs, plasmacytoid dendritic cells; DEGs, differentially expressed genes; UMAP, uniform manifold approximation and projection; Allo, allogenic group; IS, immunosuppressant; MSCs, mesenchymal stem cells; LT, liver transplantation.

pDCs were identified with 7 subpopulations defined by a set of unique genes predominantly expressed by the specific cluster in comparison with all other clusters combined, shown in **Figure 9A**. Heatmap showed the top 10 DEGs in each cluster, such as *Egr1* in pDCs_1 (**Additional Figure 5**). Among these genes, the top 3 non-overlapped genes were selected as the maker genes for each cluster, such as *Egr1*, *Fos* and *Jun* in pDCs_1 (**Figure 9B**). Particularly, the proportions of pDCs subpopulations in the Syn group significantly altered compared to other groups (**Figure 9C**).

#### 3.9.2 DEGs in pDCs subpopulations

For the Syn, Allo+IS and Allo+IS+MSCs groups, the DEGs of pDCs were analyzed between the 30 days and 60 days after LT, which have been presented in **Additional Files 21**–**23**, respectively. **Additional Files 24** and **25** showed the DEGs in pDCs between the Allo+IS+MSCs and Allo+IS, of which liver samples were collected 30 days or 60 days after LT. **Figure 9D** showed the top 10 up-regulated genes (*Ccl5*, *RT1-A1*, *Pla2g16*, *Stat1*, *RT1-A2*, *Nkg7*, *Lag3*, *AABR07017902.1*, *Cd3e* and *RT1-T24-4*) and down-regulated genes (*Ifi27*, *Clu*, *C1qa*, *Cadm3*, *C1qb*, *Krt8*, *Clec4f*, *Hp*, *Klbdc10*, and *Tmem176a*) in the Allo+IS+MSCs compared to the Allo+IS 30 days after LT. **Figure 9E** presented the top 10 up-regulated genes (*Slfn4*, *Alb*, *Kng1*, *Gzma*, *Hp*, *Hpx*, *LOC680406*, *S100a9*, *Nr4a1*, and *Pck1*) and top 10 down-regulated genes (*Cd209d*, *C1qb*, *Degs2*, *C1qc*, *Gchfr*, *Cd24*, *Lgals3*, *Gpnmb*, *Hmox1*, and *Lgmn*) in the Allo+IS+MSCs compared to the Allo+IS 60 days after LT. These DEGs may be implicated in regulating antigen processing and presentation, complement and coagulation cascades, allograft rejection, and oxidative phosphorylation in pDCs (**Figures 9F–I**).

### 3.10 Neutrophils subpopulations in liver tissues and comparison of genes in each sample

#### 3.10.1 Neutrophils subpopulations

**Figure 10:**
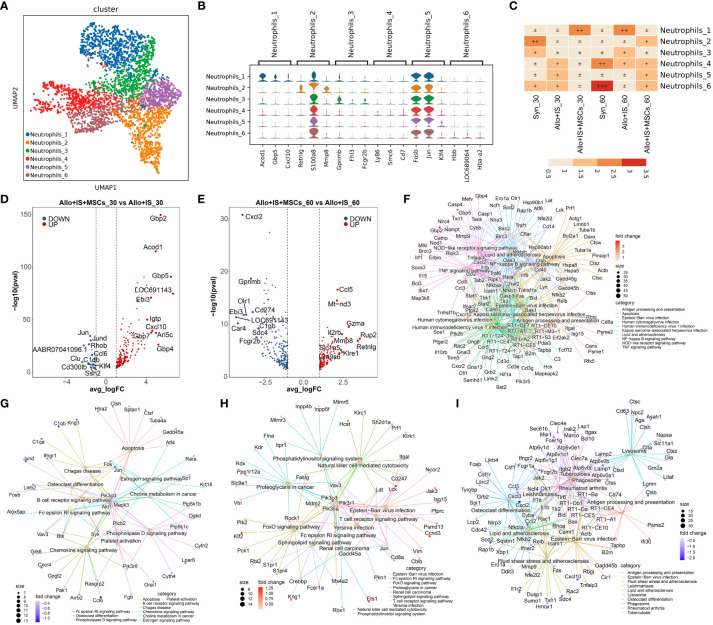
Identifying neutrophils subclusters and analysis of DEGs. **(A)** UMAP plots showing the distribution of 6 neutrophils subpopulations. **(B)** Dot plots suggesting the top 3 highly expressed genes (x-axis) of 6 predominant subpopulations (y-axis); The size of the dot indicates the fraction of cell subpopulation and the color of the dots is the expression level of the marker genes. **(C)** Heatmap presenting the proportion of 6 neutrophils subtypes in each sample. Volcano plots showing the top 10 differentially expressed genes between the Allo+IS and Allo+IS+MSCs 30 days **(D)** or 60 days **(E)** after LT. Net plots showed the functional analysis based on the up-regulated genes **(F)** or down-regulated genes **(G)** of neutrophils between the Allo+IS+MSCs and Allo+IS 30 days after LT, and up-regulated genes **(H)** or down-regulated genes **(I)** between the Allo+IS+MSCs and Allo+IS 60 days after LT. DEGs, differentially expressed genes; UMAP, uniform manifold approximation and projection; Allo, allogenic group; IS, immunosuppressant; MSCs, mesenchymal stem cells; LT, liver transplantation.

Six subpopulations (neutrophils_1, neutrophils_2, neutrophils_3, neutrophils_4, neutrophils_5, and neutrophils_6) were identified in neutrophils, and the distribution was shown in **Figure 10A**. **Additional Figure 6** presented the top 10 feature genes in each neutrophil subpopulation, such as *Acod1* in neutrophils_1 subcluster. Among these, violin plots representatively exhibited the expression of the top 3 genes in the specified subpopulation, including neutrophils_1 (*Acod1*, *Gbp5*, *Cxcl10*), neutrophils_2 (*Retnlg*, *S100a8*, *Mmp8*), neutrophils_3 (*Gpnmb*, *Fhl3*, *Fcgr2b*), neutrophils_4 (*Ly86*, *Smc6*, *Cd7*), neutrophils_5 (*Fosb*, *Jun*, *Klf4*), and neutrophils_6 (*Hbb*, *LOC689064*, *Hba-a2*) (**Figure 10B**). **Figure 10C** indicated that the composition of neutrophils was altered by MSCs 30 or 60 days after LT, compared to the Allo+IS group. Of note, in the syn group, the proportions of neutrophils_4 and neutrophils_6 increased 60 days after LT compared to 30 days after LT, which was comparable to the Allo+IS+MSCs group and opposite to the Allo+IS group.

#### 3.10.2 DEGs in neutrophils subpopulations

**Additional File 26** listed the DEGs of neutrophils subpopulations in the Syn group between 30 days post-LT and 60 days post-LT. The DEGs between 30 days and 60 days after LT in the Allo+IS and Allo+IS+MSCs were presented in **Additional Files 27** and **28**, respectively. Next, we compared the DEGs between the Allo+IS and Syn 30 days after LT (**Additional File 29**) and 60 days after LT (**Additional File 30**). Further, we especially compared the DEGs between the Allo+IS+MSCs and Allo+IS groups, 30 days and 60 days after LT. The top 10 up-regulated (*Gbp2*, *Acod1*, *Gbp5*, *LOC691143*, *Ebi3*, *Igtp*, *Cxcl10*, *Gbp7*, *Arl5c*, *Gbp4*) or down-regulated (*Jun*, *Jund*, *Rhob*, *AABR07041096.1*, *Ccl6*, *Clu*, *Clqb*, *Cd300lb*, *Klf4*, *Ssh2*) genes were compared between the Allo+IS and Allo+IS+MSCs 30 days after LT (**Figure 10D**). As for 60 days after LT, **Figure 10E** showed the top 10 up-regulated (*Ccl5*, *Mt-nd3*, *Gzma*, *Il2rb*, *Mmp8*, *Rup2*, *Retnlg*, *Slc1a5*, *Klre1*, *Ly49s6*) and top 10 down-regulated (*Cxcl2*, *Gpnmb*, *Cd274*, *Ebi3*, *Olr1*, *LOC691143*, *Car4*, *C1qb*, *Sdc4*, *Fcgr2b*) genes between the Allo+IS+MSCs and Allo+IS. At 30 days after LT, the application of MSCs increased the expression of *LOC691143*, *Ebi3*, and *Acod1*, suggesting the accumulation of neutrophil_6, compared to the Allo+IS group. Further, the expression of *LOC691143* and *Ebi3* decreased in the Allo+IS+MSCs compared to the Allo+IS group 60 days after LT, indicating that the prolonged application of MSCs may reduce the proportion of neutrophils to a certain extent. At 30 days and 60 days after LT, the DEGs between the Allo+IS+MSCs and Allo+IS participated in regulating antigen processing and presentation, B cell receptor signaling pathway, natural killer cell mediated cytotoxicity, and lysosome (**Figures 10F–I**).

## 4 Discussion

The liver has a unique composition of parenchymal and immune cells that regulate innate and adaptive immunity and promote antigen-specific tolerance (52). Although the mechanisms underlying liver transplant tolerance are not well understood, important insights have been gained into how the local microenvironment, hepatic immune cells, and specific molecular pathways can promote donor-specific tolerance (53–55). The application of MSCs in the management of transplant rejection and inflammatory scenario is of particular interest due to their ability to mediate the biological responses of immune cells implicated in these courses (56, 57). Using single-cell sequencing technique, this study is the first to evaluate the biological and transcriptomic characterization of immune cells in LT rats receiving MSCs under classical immunosuppressant agents-based immunosuppression.

The acceptance of completely MHC-mismatched (fully allogeneic) orthotopic liver transplants in the absence of immunosuppressive therapy in rodents is well-recognized (28, 58). In rats, indefinite liver allograft survival in the absence of any immunosuppressive drug therapy is also strain-dependent and related to MHC (RT1 in rats) disparity (28, 58). Our results indicated that the presence of immunosuppressive therapy induced the acceptance of orthotopic liver transplants in Lewis rats, instead of in combination with MSCs, which were evidenced by the physiological and laboratory indexes. Conversely, the efficacy of the Allo+IS+MSCs was superior to that of the Allo+IS in the early stage of LT within 15 postoperative days. A phase I, prospective, controlled study reported that MSCs infusion confers no side effect 3 days after LT and did not promote tolerance (59). In contrast, a phase I/II randomized, open-label, controlled trial has indicated that MSCs may be introduced as a novel immunosuppressive approach for ABO-incompatible LT due to its comparable results to rituximab and prevention of infection and biliary complication (60). Schacher et al. confirmed that infusion of BM-derived MSCs is feasible for the treatment of patients with acute-on-chronic liver failure at grades 2 and 3 without infusion-related side effects (61). This seemingly paradoxical finding can be explained by the fact that serving as an immunomodulator, MSCs may play a bidirectional regulatory role in immunity. When inflammation level is high *in vivo*, MSCs inhibit the inflammatory response; when inflammation level is low *in vivo*, MSCs may act as a pro-inflammatory agent (62).

In the process of LT, immune cells from the recipient enter the donor liver to reshape a new immune microenvironment together with the resident immune cells (63). The application of immunosuppressant may inhibit immune responses through decreasing the proportion of B cells and neutrophil, while may induce liver injury by decreasing cholangiocytes, endothelial cells, hepatic stellate cells, and hepatocytes (64, 65), which has also observed in our study. Neutrophils have been associated with liver ischemia-reperfusion injury in LT (66). pDCs may weekly stimulate T cell responses and play a part in the induction of liver transplant tolerance (67). Liver resident KCs help to restore tissue integrity following injury, but can also contribute to liver disease progression (68). Hepatic stellate cells are crucial for hepatic wound repair and tissue modelling, but they might also have an important role in maintenance of immune homeostasis and inherent liver tolerogenicity (69). Here, through examination of the immune cell composition and gene expression, we elucidated the liver immunobiology that underpin our current understanding of liver allograft tolerance affected by immunosuppressant alone or in combination with MSCs. These findings presented the immunomodulatory roles of immunosuppressant combined with MSCs after LT, which may explain the functional mechanism of immunosuppressive agents on immune cells and marker genes. The obtained immune cells and marker genes can be designed for targeted therapy.

MSCs are multipotent progenitors, capable of differentiating into various cells and regulating immune responses (70, 71). A large number of *in vitro* and *in vivo* studies have documented the anti-inflammatory and immunoregulatory properties of MSCs on both the adaptive and innate immune system, as well as a potential beneficial effect in ischemia–reperfusion injury (72–74). Specifically, MSCs have been shown to decrease effector T cell response while promoting the emergence of Treg (75). These MSC properties suggested that they could be particularly attractive in solid organ transplantation. With single-cell RNA sequencing, we have identified the intrahepatic cell populations of parenchymal cells (hepatocytes), non-parenchymal cells (endothelial cells and cholangiocytes), hepatic stellate cells, liver resident and infiltrating lymphocytes (B cells and T cells), MPs, antigen-presenting cells, and granulocytes (neutrophils). The application of immunosuppressant alone or combination with MSCs led to the loss of fragile cells (hepatocytes, endothelial cells, and cholangiocytes) 60 days post LT, and not significantly inhibit inflammatory reaction. Therefore, we considered that the inhibitory effects of MSCs on immunity can be utilized to suppress inflammation reaction in the early stages of LT.

We annotated the liver-resident T cells subpopulations, which majorly includes CD8+ Teff, Tex, Th, NKT, naïve T cells, and Treg. The application of the immunosuppressants or MSCs distinctly modifies the proportion of the subsets of T cells compared to the Allo group, particularly, Mki67+CD8+ T cells, CD8+ Teff expressing *Gzmk*, *Xcl1*, or *Lag3*, Th cells expressing *Ccr6*, *Cd40lg*, or *Tnfrsf4*, NK cells expressing *Fcer1g*, *Gzmk*, *Itgax*, or *Xcl1*, Lef1+ naïve T cells, and foxp3+ Treg cells. Jonsson et al. demonstrated that *Gzmk*-expressing CD8+ T cells are the major CD8+ T cell subsets in human tissues, showing the potential to drive inflammation (76). The increased proportion of Gzmk+ CD8+ T cells has been found in transplanted liver with mild rejection (77) or in kidney transplantation with subclinical and acute cellular rejection (78). Xcl1 belongs to C class chemokine, which is generally expressed by T, NK and NKT cells during infectious and inflammatory responses (79). Xcl1+CD8+ Teff and Xcl1+ NK cells were altered inversely after LT, and MSCs may inhibit the proportion of Xcl1+ NK in particular. The immune checkpoint receptor Lag3 was expressed by the most T cell types, which was one of the most promising inhibitory receptor targets in clinical practice (80). Foxp3+CD4+CD25+ Treg cells appear to underpin spontaneous acceptance of major histocompatibility complex-mismatched liver allografts in mice (81). Accordingly, through monitoring the proportion of intrahepatic immune T cells (Lag3+CD8+ Tex, Foxp3+ Treg, Gzmk+ NK cells), T cells-mediated immune infiltration or reaction could be reflected after LT or application of immunosuppressant or MSCs. Besides, the marker gene *Lag3* could be targeted to inhibit excessive immune reaction induced by LT.

After 30 days and 60 days post LT, the composition of T cell subsets changed, and the alterations of different T cell subsets may be related to the up-regulation and down-regulation of functional gene. GO analysis suggested that the vast majority of the down-regulated genes may involve in signal transduction in external side of plasma membrane, positive regulation of cytokine production, adaptive immune response based on somatic recombination of immune receptors built from immunoglobulin superfamily domains, lymphocyte migration, regulation of peptidase and hydrolase, migration of leukocyte and mononuclear, as well as differentiation of mononuclear cells and lymphocytes. The down-regulated genes primarily mediated cytokine binding, leukocyte cell-cell adhesion, and ribosome constituent. Immunosuppressant alone or in combination with MSCs inhibiting chronic immune-mediated liver damages may be due to their affection on gene expression and intercellular interaction of immune cells. Cumulative data have revealed the extrathymic pathway of T cell differentiation, such as in the hepatic sinusoids (82, 83). Pseudotime analysis revealed the transitional states of T cells after LT, beginning at the NK or Treg, transiting into Th or CD8+ Teff, next differentiating into CD8+ Tex or naïve T cells, and finally ending at proliferating T cells. Our study is one of the first to characterize the transition of T cells at two interval times after LT in detail. Specially, Tregs were predominantly induced after exposure to immunosuppressant, while naïve T cells were not significantly observed both in the Allo+IS and Allo+IS+MSCs groups. Our data suggested that gene expression in T cells was altered along a trajectory of pseudotime, such as *Birc5*, *Ccl4*, *Ccl5*, *Cd7*, *Cdkn3, Cenpe*, *Cenpf*, and *Fcer1g*.

Monocytes are a subset of circulating mononuclear leukocytes involving in maintaining tissue homeostasis and mounting immune responses (84). Human monocytes are subdivided into three main subsets: classical (CD14+, CD16-), non-classical (CD14^dim^, CD16+) and intermediate (CD14+, CD16+) (85). Classical monocytes secrete higher pro-inflammatory cytokines during infection and are likely to play roles in inflammation, whereas non-classical monocytes are believed to produce higher anti-inflammatory cytokines and are considered to be involved in repair process (86). We compared the proportions of classical and non-classical monocytes from the liver tissues of the Allo+IS and Allo+IS+MSCs groups. The results showed that 60 days post liver transplantation, immunosuppressant in combination with MSCs significantly reduced the proportion of classical monocytes and increased the proportion of non-classical monocytes compared to the Allo+IS group. This finding implied that immunosuppressant in combination with MSCs may suppress alloimmune responses by acting on classical monocytes and non-classical monocytes. *Fn1* encodes fibronectin, involved in cell adhesion, migration and growth, known to be specifically upregulated in inflammatory monocytes (87). *IFITM3*, localizing in endolysosomes, is essential for innate defense against influenza virus in mice and human (88). *Eno3* has been recently reported to be up-regulated in non-alcoholic fatty liver disease and regulate ferroptosis and lipid accumulation (89), however, its role in immune reaction has not been studied. Our data indicated that, in intrahepatic monocytes, classical monocytes characteristically expressed *Fn1* and *Ifitm3*, and non-classical monocytes expressed *Fn1*, *IFITM3*, and *Eno3*. *Fn1-* and *Ifitm3*- expressing classical monocytes and *Eno3*-expressing non-classical monocytes may function in response to immunological rejection in LT. Here, we considered that intrahepatic Fn1+Ifitm3+ classical monocytes may be a maker immune cell during the inflammation or infection after LT.

Macrophages, derived from monocytes, were classified into 8 subgroups including the resident macrophages Kupffer cell in the liver in our study. Macrophages possesses three main functions in both innate and adaptive immune system, including phagocytosis, antigen presentation and cytokine production, which play a pivotal role in triggering and sustaining the sterile inflammation during in ischemia-reperfusion injury (90). Our results indicated that the proportions of the subgroups macrophages_1 was particularly decreased 60 days post allogeneic LT. The subgroup macrophages_1 was characterized by the expression of *Mmp12*, *Trpc6*, and *Gpr183*. Macrophage-derived metalloelastase 12 encoding by *Mmp12* appears to mediate elastin degradation that has been linked to maturity of liver fibrosis (91). It has been indicated that receptor channel Trpc6 orchestrates the activation of human hepatic stellate cell under hypoxia condition (92). Studies have elucidated that the orphan G protein-coupled receptor GPR183 expressed by activated B cells is essential for the guidance of B cells moving to extrafollicular sites and the induction of early plasmablast responses (93). KEGG pathway analysis revealed that the feature genes of macrophages may play roles in cell adhesion, adherens junction, complement and coagulation, and PPAR signaling pathway to achieve the above functions. Consequently, the proportion of macrophages and the corresponding marker gene *Mmp12* may be used as indicators to reveal immune reaction or targets to inhibit excessive inflammation reactions caused by LT.

In the steady-state blood circulation, neutrophils are dominant immune cells (94, 95). Neutrophils are recruited to the injury site in ischemia-reperfusion-stressed blood-perfused liver, leading to sterile inflammation and contributing to the hepatocellular damage (96, 97). Therefore, in addition to being considering as innate effector cells, neutrophil infiltration into hepatic sinusoidal lumen is also recognized as a reliable biomarker of liver ischemia-reperfusion injury (96, 97). Obviously, immunosuppressant or in combination with MSCs altered the proportions of the 6 subgroups. These clusters were characterized by several genes such as *S100a8*, *Fosb*, *Jun* and *Klf4*. Immunological properties of S100 proteins have been clarified in activated neutrophils and macrophages (98). It is noteworthy that early acute cellular rejection within 90 days of LT showed significant changes in *Fosb* expression, which may serve as a predictive signature (99). Thus, the function of MSCs and immunosuppressants are closely related to the effects of *S100a8*, *Fosb*, *Jun* and *Klf4*. Monitoring of molecule set could distinguish between tolerance and rejection. The mechanisms that underlie the induction and maintenance of liver transplant tolerance, and that determine whether immunosuppressive therapy can be safely withdrawn, are poorly understood. Besides, there are even no validated biomarkers that can reliably predict rejection or tolerance. However, several molecules like cytokines, microRNAs, or inflammatory genes have been suggested as potential biomarkers of tolerance or rejection. Of particular, specific sets of genes such as those encoding FOXP3, PD1, PDL1 and TIM3 have been associated with tolerance and successful withdrawal of immunosuppressive drugs.

Nevertheless, there are many unexplained issues in this study. The negligible effect of MSCs combined with immunosuppressant may be due to insufficient sample size, immunosuppressant-based regimen, or insufficient dose of MSCs, which may need to be increased or adjusted appropriately. In addition, this study did not assess the effect of the stage of MSCs infusion (preoperative, intraoperative or postoperative) and the mode of infusion (peripheral vein, portal vein or hepatic artery) on immune cells, which may be important influencing factors. Moreover, the immunosuppressive effect of MSCs may be related to their source (adipose tissue, bone marrow, and liver tissues) or donors (organ donor or recipient).

## 5 Conclusions

Overall, our study firstly delineated the distinct immune subsets of intrahepatic liver transplant cells. Of particular, we annotated the subpopulations of immune cell types and as well as their dynamic alterations. From single-cell resolution, we better understand the heterogeneity and subpopulations of T cells, MPs, classical monocytes, non-classical monocytes, pDCs, and neutrophils, caused by the application of immunosuppressant alone or in combination with MSCs. The hepatocytes and the corresponding markers (*AABR07034632.1*, *Sult1c3*, *Rup2*) can be used to indicate the damage of liver tissue. The immune response after LT may be suggested by the proportion of B cells, neutrophils, T cells, and macrophages, as well as their marker genes (*AABR07034632.1*, *Sult1c3*, *Rup2* for hepatocytes; *Ms4a1* and *Cd79b* for B cells; *S100a9*, *Il1r2*, and *Ifit1bl* for neutrophils; *Lag3* for Lag3+CD8+ Tex, Foxp3+ Treg, Gzmk+ NK cells; *Mmp12* for macrophages). Further, the functional contributions of immune cells were altered by the immunosuppressant and MSCs. Our results help to ascertain immune cells to indicate the immune reaction caused by LT and provide novel therapeutic targets to design immunosuppressive drugs, which may assist in inhibiting liver allograft rejection for patients receiving LT.

These findings may help to ascertain novel therapeutic targets to inhibit rejection after LT.

## Data availability statement

The datasets presented in this study can be found in online repositories. The names of the repository/repositories and accession number(s) can be found below: CRA012066 (https://ngdc.cncb.ac.cn/search/?dbId=gsa&q=CRA012066).

## Ethics statement

The animal study was approved by Animal Ethics Committee of Mengchao Hepatobiliary Hospital of Fujian Medical University (NO. MCHH-AEC-2023-04-01). The study was conducted in accordance with the local legislation and institutional requirements.

## Author contributions

HTL: Conceptualization, Data curation, Funding acquisition, Methodology, Supervision, Writing – original draft, Writing – review & editing. SY: Conceptualization, Data curation, Investigation, Methodology, Visualization, Writing – original draft. HYL: Formal Analysis, Methodology, Resources, Validation, Writing – original draft. LC: Data curation, Investigation, Methodology, Visualization, Writing – original draft. HZL: Data curation, Methodology, Writing – original draft, Software. XL: Formal Analysis, Methodology, Visualization, Writing – original draft. CS: Methodology, Validation, Writing – original draft.
