# Supplementary figures and images for: Immunologic barriers in liver transplantation: a single-cell analysis of the role of mesenchymal stem cells
Source: Front Immunol. 2023 Dec 7;14:1274982. doi: 10.3389/fimmu.2023.1274982 (PMC10748593; doi:10.3389/fimmu.2023.1274982)

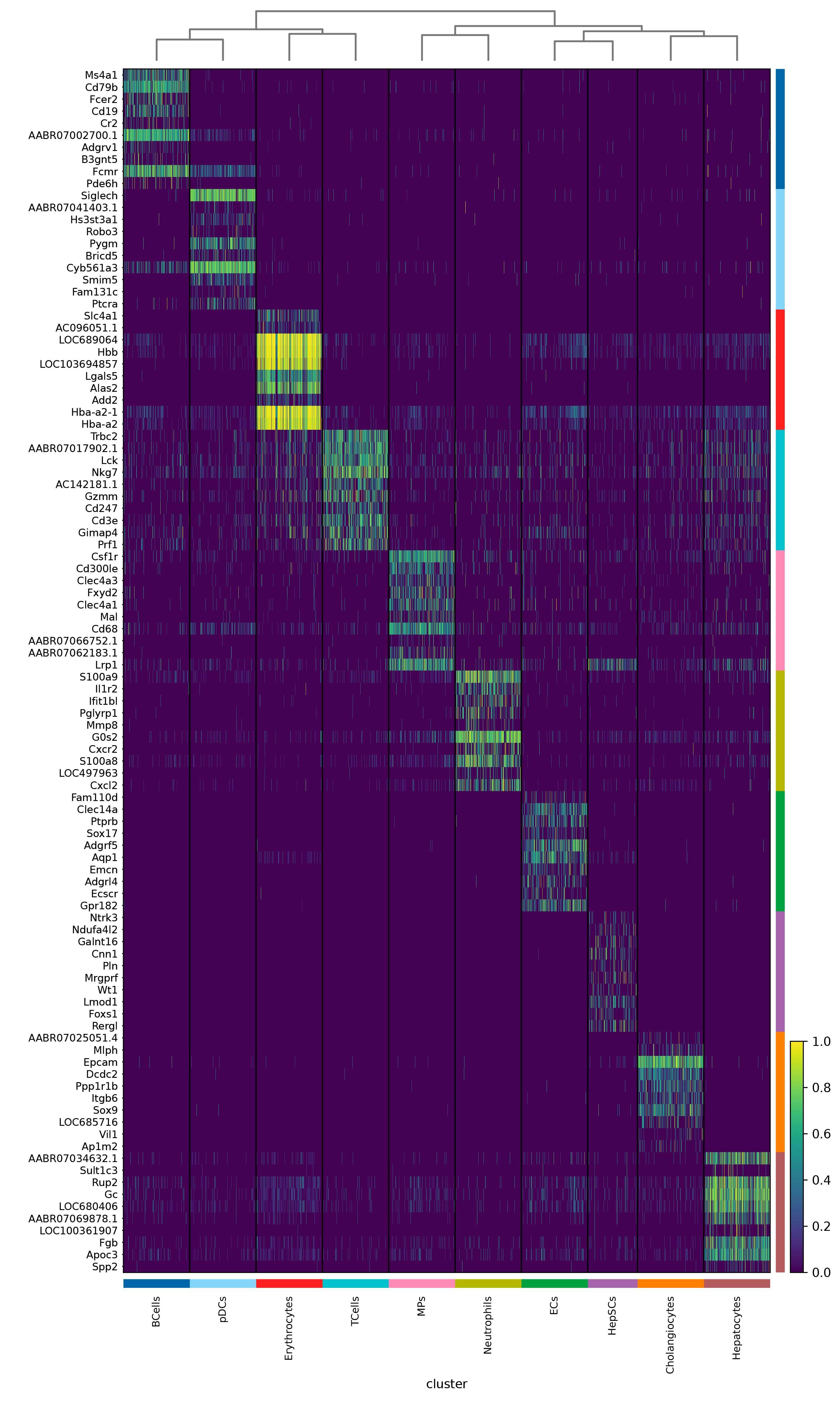

Supplement: Supplementary file 1 [file DataSheet_1.zip › Additional Figure 1.tif]

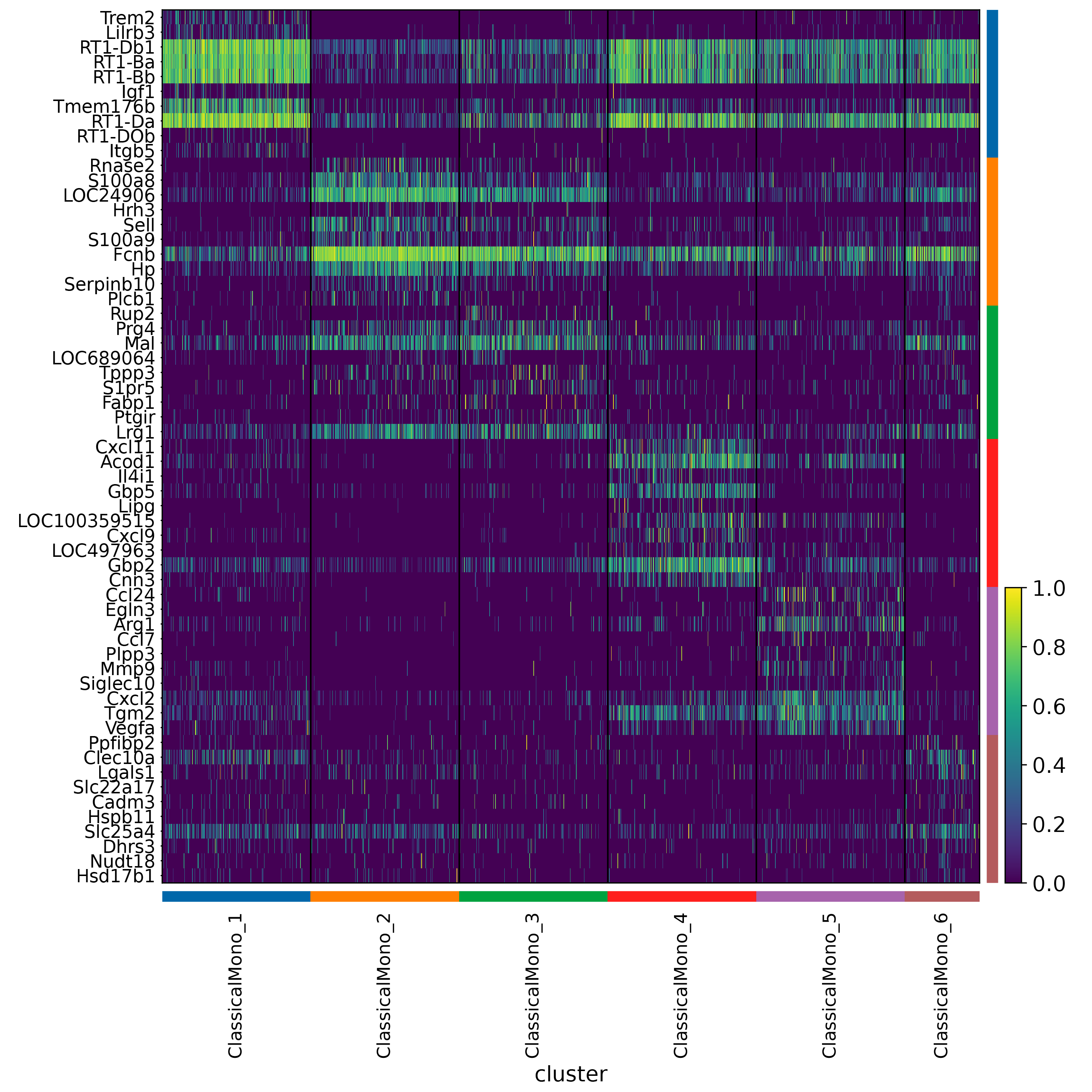

Supplement: Supplementary file 1 [file DataSheet_1.zip › Additional Figure 2.tif]

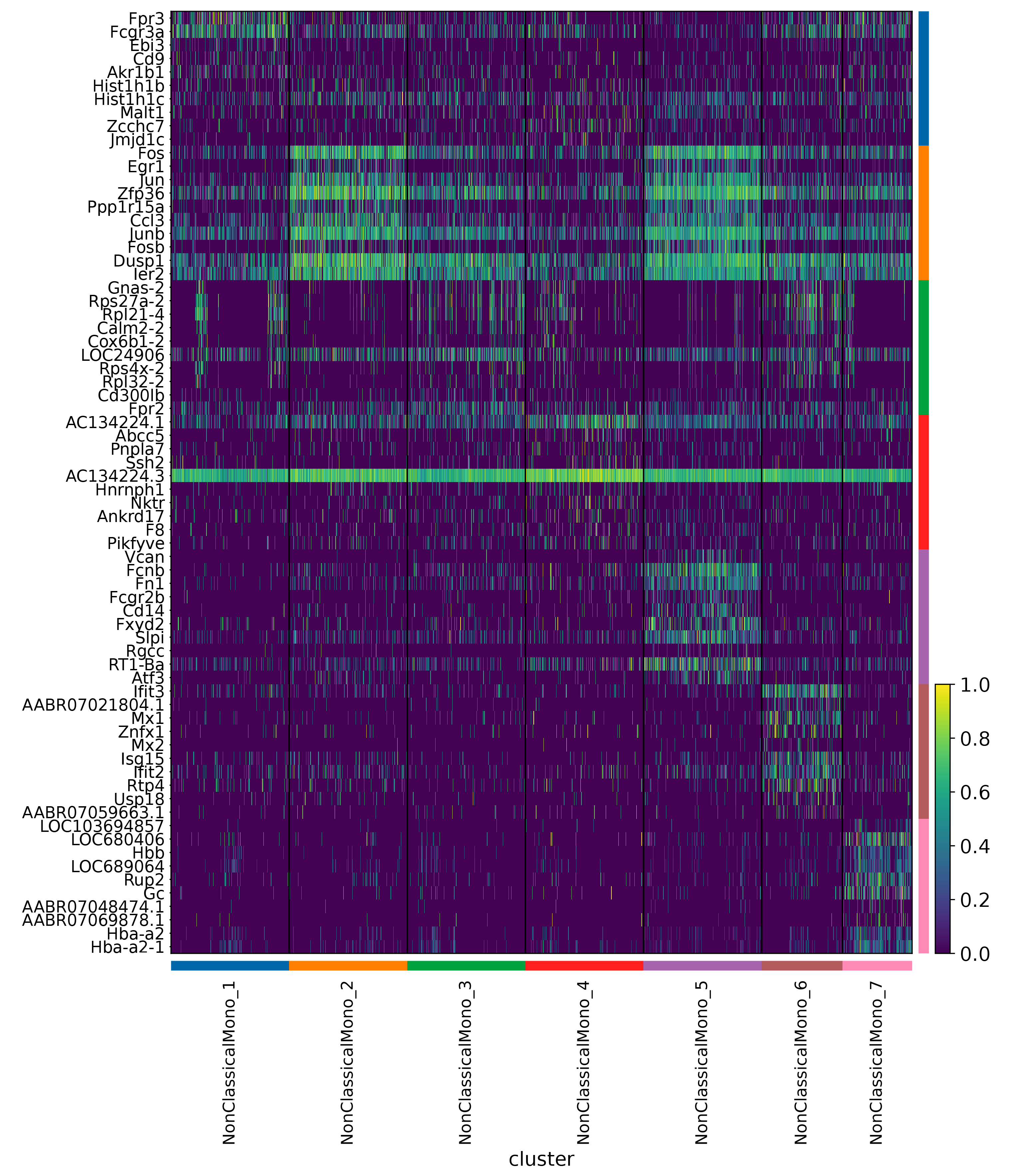

Supplement: Supplementary file 1 [file DataSheet_1.zip › Additional Figure 3.tif]

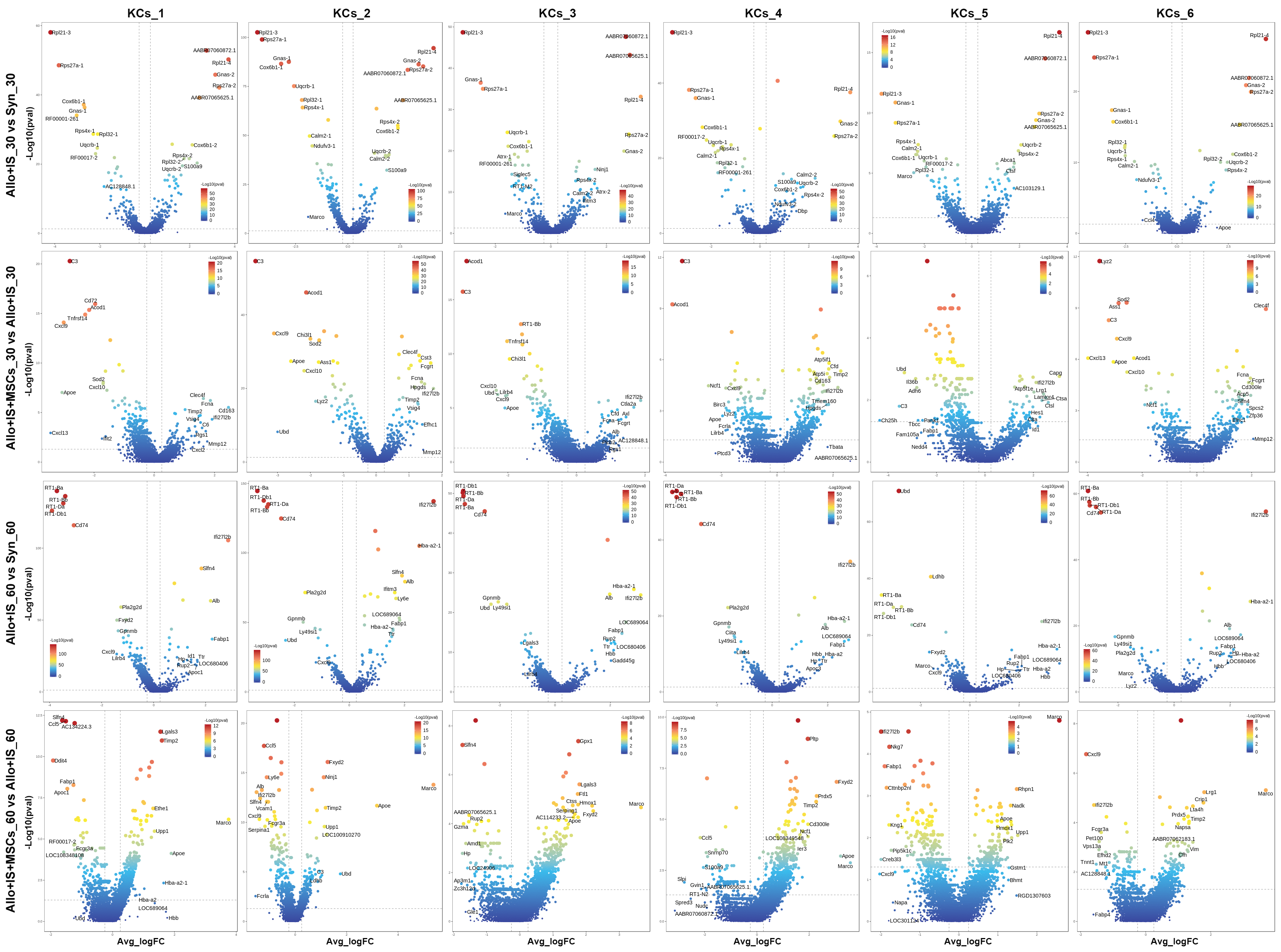

Supplement: Supplementary file 1 [file DataSheet_1.zip › Additional Figure 4.tif]

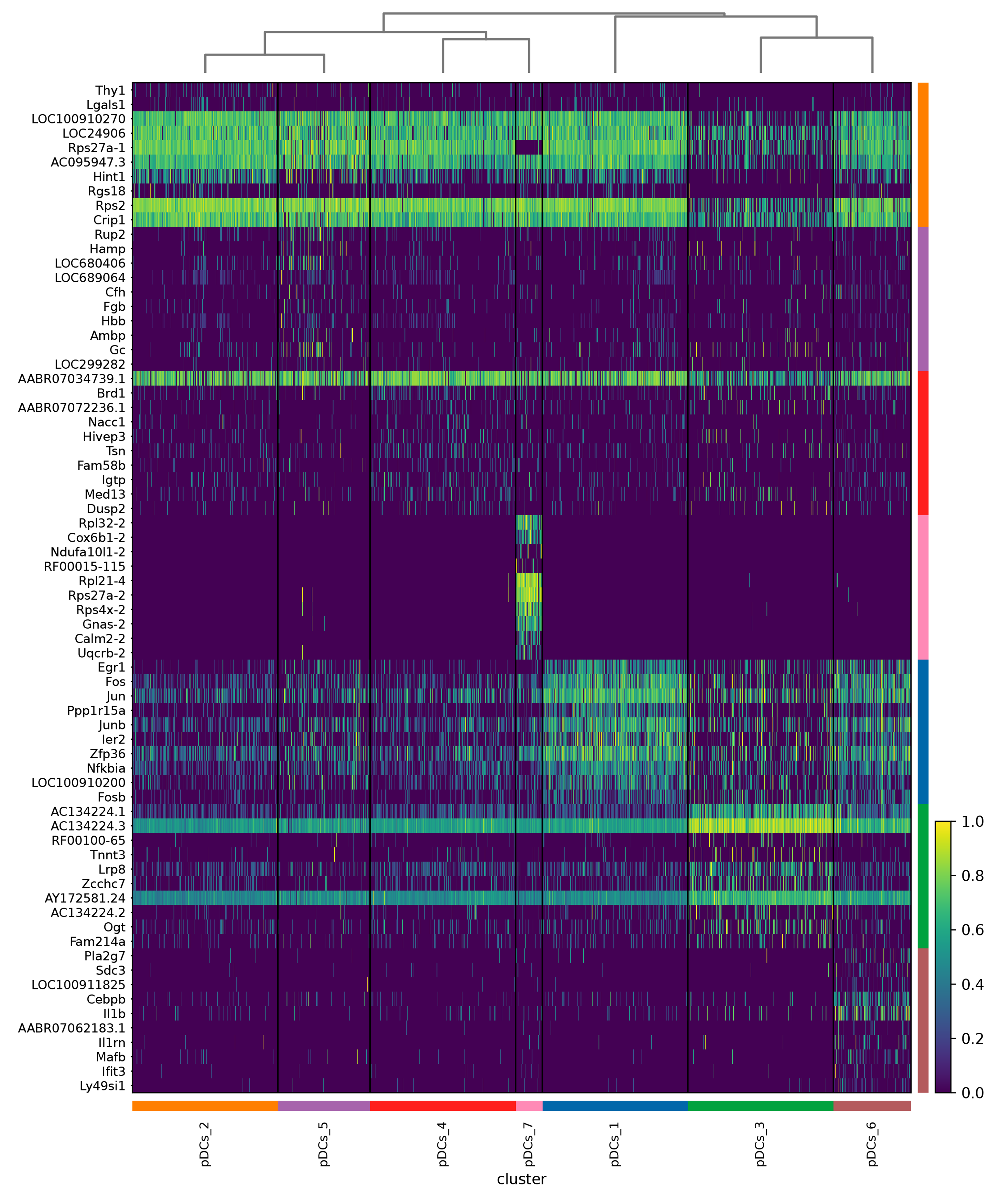

Supplement: Supplementary file 1 [file DataSheet_1.zip › Additional Figure 5.tif]

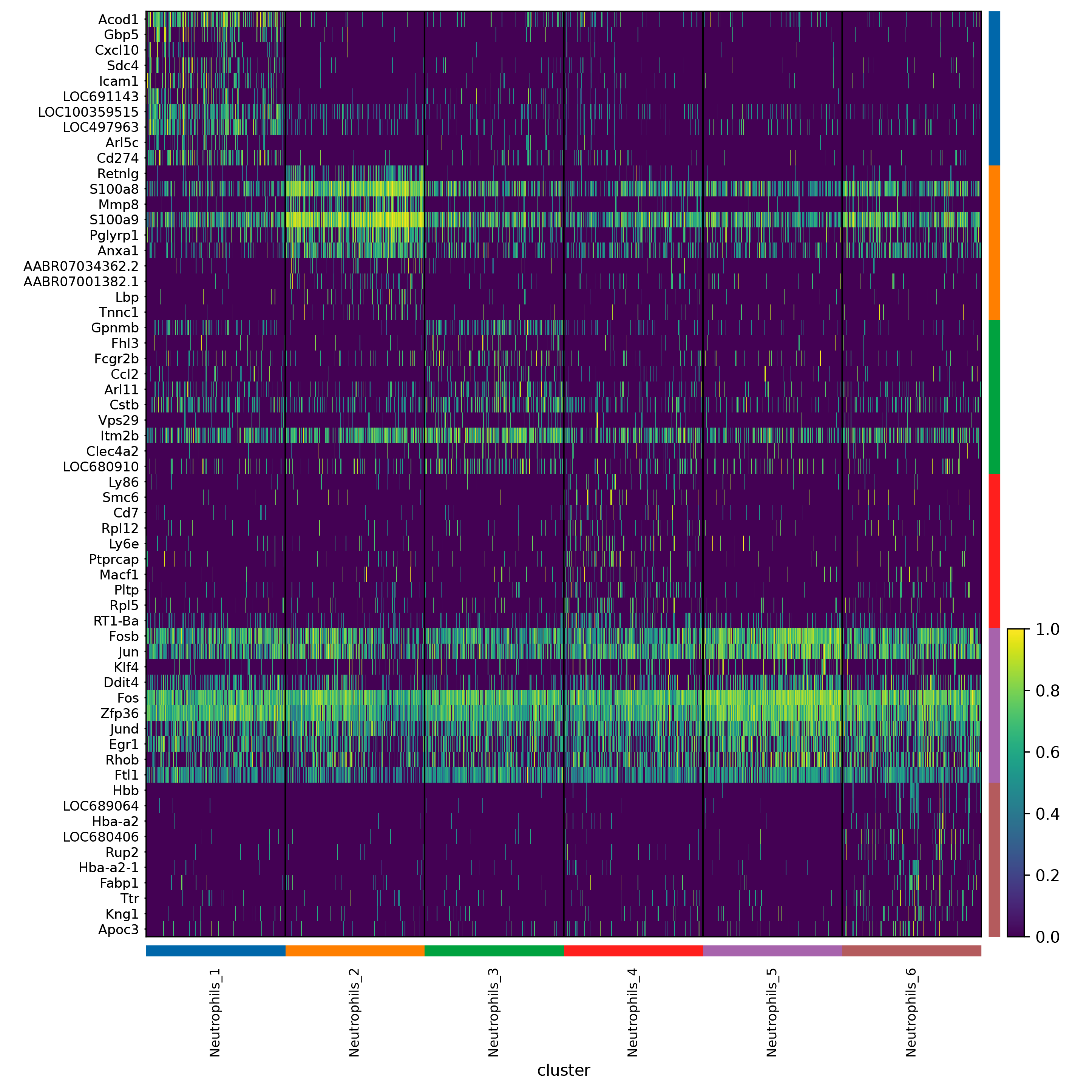

Supplement: Supplementary file 1 [file DataSheet_1.zip › Additional Figure 6.tif]
