# Supplementary material for: Immunologic barriers in liver transplantation: a single-cell analysis of the role of mesenchymal stem cells
Source: Front Immunol. 2023 Dec 7;14:1274982. doi: 10.3389/fimmu.2023.1274982 (PMC10748593; doi:10.3389/fimmu.2023.1274982)
Supplement: Supplementary file 2 [file DataSheet_2.docx]

**Additional Figure 1 Heatmap of top 10 DEGs (y-axis) between different clusters of intrahepatic cells (x-axis) from pooled liver samples.** DEGs, differentially expressed genes.

**Additional Figure 2 Heatmap of top 10 DEGs (y-axis) between different clusters of classical monocytes subclusters (x-axis) from pooled liver samples.** DEGs, differentially expressed genes.

**Additional Figure 3 Heatmap of top 10 DEGs (y-axis) between different clusters of non-classical monocytes subclusters (x-axis) from pooled liver samples.** DEGs, differentially expressed genes.

**Additional Figure 4 Volcano plats presenting the top 10 DEGs of KCs subclusters between the Allo+IS and Syn or Allo+IS+MSCs and Allo+IS 30 days or 60 days after LT.** DEGs, differentially expressed genes.

**Additional Figure 5 Heatmap of top 10 DEGs (y-axis) between different clusters of pDCs subclusters (x-axis) from pooled liver samples.** DEGs, differentially expressed genes.

**Additional Figure 6 Heatmap of top 10 DEGs (y-axis) between different clusters of neutrophils subclusters (x-axis) from pooled liver samples.** DEGs, differentially expressed genes.

**Additional File 1** Comparison of differentially expressed genes in T cells for the Syn group between 30 days and 60 days after LT.

**Additional File 2** Comparison of differentially expressed genes in T cells for the Allo+IS group between 30 days and 60 days after LT.

**Additional File 3** Comparison of differentially expressed genes in T cells for the Allo+IS+MSCs group between 30 days and 60 days after LT.

**Additional File 4** Comparison of differentially expressed genes in T cells between the Allo+IS and Syn 30 days after LT.

**Additional File 5** Comparison of differentially expressed genes in T cells between the Allo+IS and Syn 60 days after LT.

**Additional File 6** Comparison of differentially expressed genes in MPs for the Syn group between 30 days and 60 days after LT.

**Additional File 7** Comparison of differentially expressed genes in MPs for the Allo+IS group between 30 days and 60 days after LT.

**Additional File 8** Comparison of differentially expressed genes in MPs for the Allo+IS+MSCs group between 30 days and 60 days after LT.

**Additional File 9** Comparison of differentially expressed genes in MPs between the Allo+IS and Syn 30 days after LT.

**Additional File 10** Comparison of differentially expressed genes in MPs between the Allo+IS and Syn 60 days after LT.

**Additional File 11** Comparison of differentially expressed genes in classical monocytes for the Syn group between 30 days and 60 days after LT.

**Additional File 12** Comparison of differentially expressed genes in classical monocytes for the Allo+IS group between 30 days and 60 days after LT.

**Additional File 13** Comparison of differentially expressed genes in classical monocytes for the Allo+IS+MSCs group between 30 days and 60 days after LT.

**Additional File 14** Comparison of differentially expressed genes in classical monocytes between the Allo+IS and Syn 30 days after LT.

**Additional File 15** Comparison of differentially expressed genes in classical monocytes between the Allo+IS and Syn 60 days after LT.

**Additional File 16** Comparison of differentially expressed genes in non-classical monocytes for the Syn group between 30 days and 60 days after LT.

**Additional File 17** Comparison of differentially expressed genes in non-classical monocytes for the Allo+IS group between 30 days and 60 days after LT.

**Additional File 18** Comparison of differentially expressed genes in non-classical monocytes for the Allo+IS+MSCs group between 30 days and 60 days after LT.

**Additional File 19** Comparison of differentially expressed genes in non-classical monocytes between the Allo+IS and Syn 30 days after LT.

**Additional File 20** Comparison of differentially expressed genes in non-classical monocytes between the Allo+IS and Syn 60 days after LT.

**Additional File 21** Comparison of differentially expressed genes in plasmacytoid dendritic cells (pDCs) for the Syn group between 30 days and 60 days after LT.

**Additional File 22** Comparison of differentially expressed genes in plasmacytoid dendritic cells (pDCs) for the Allo+IS group between 30 days and 60 days after LT.

**Additional File 23** Comparison of differentially expressed genes in plasmacytoid dendritic cells (pDCs) for the Allo+IS+MSCs group between 30 days and 60 days after LT.

**Additional File 24** Comparison of differentially expressed genes in plasmacytoid dendritic cells (pDCs) between the Allo+IS and Syn 30 days after LT.

**Additional File 25** Comparison of differentially expressed genes in plasmacytoid dendritic cells (pDCs) between the Allo+IS and Syn 60 days after LT.

**Additional File 26** Comparison of differentially expressed genes in neutrophils for the Syn group between 30 days and 60 days after LT.

**Additional File 27** Comparison of differentially expressed genes in neutrophils for the Allo+IS group between 30 days and 60 days after LT.

**Additional File 28** Comparison of differentially expressed genes in neutrophils for the Allo+IS+MSCs group between 30 days and 60 days after LT.

**Additional File 29** Comparison of differentially expressed genes in neutrophils between the Allo+IS and Syn 30 days after LT.

**Additional File 30** Comparison of differentially expressed genes in neutrophils between the Allo+IS and Syn 60 days after LT.
